# A chromosomal-level genome assembly for the insect vector for Chagas disease, *Triatoma rubrofasciata*

**DOI:** 10.1093/gigascience/giz089

**Published:** 2019-08-19

**Authors:** Qin Liu, Yunhai Guo, Yi Zhang, Wei Hu, Yuanyuan Li, Dan Zhu, Zhengbin Zhou, Jiatong Wu, Nansheng Chen, Xiao-Nong Zhou

**Affiliations:** 1 National Institute of Parasitic Diseases, Chinese Center for Disease Control and Prevention; Key Laboratory of Parasite and Vector Biology, Ministry of Health; WHO Collaborating Center for Tropical Diseases; Chinese Center for Tropical Diseases Research, Shanghai 200025, P. R. China; 2 Department of Microbiology and Microbial Engineering, School of Life Sciences, Fudan, Shanghai 200025, P. R. China; 3 CAS Key Laboratory of Marine Ecology and Environmental Sciences, Institute of Oceanology, Chinese Academy of Sciences, Qingdao, Shandong 266071, P. R. China; 4 Laboratory for Marine Ecology and Environmental Science, Qingdao National Laboratory for Marine Science and Technology, Qingdao, Shandong 266237, P. R. China; 5 Department of Molecular Biology and Biochemistry, Simon Fraser University, Burnaby, British Columbia, Canada

**Keywords:** *Triatoma rubrofasciata*, PacBio Sequel platform, Hi-C, chromosomal-level assembly, comparative genomics, RNA-Seq, Iso-Seq

## Abstract

**Background:**

*Triatoma rubrofasciata* is a widespread pathogen vector for Chagas disease, an illness that affects approximately 7 million people worldwide. Despite its importance to human health, its evolutionary origin has not been conclusively determined. A reference genome for *T. rubrofasciata* is not yet available.

**Finding:**

We have sequenced the genome of a female individual with *T. rubrofasciata*using a single molecular DNA sequencing technology (i.e., PacBio Sequel platform) and have successfully reconstructed a whole-genome (680-Mb) assembly that covers 90% of the nuclear genome (757 Mb). Through Hi-C analysis, we have reconstructed full-length chromosomes of this female individual that has 13 unique chromosomes (2n = 24 = 22 + X1 + X2) with a contig N50 of 2.72 Mb and a scaffold N50 of 50.7 Mb. This genome has achieved a high base-level accuracy of 99.99%. This platinum-grade genome assembly has 12,691 annotated protein-coding genes. More than 95.1% of BUSCO genes were single-copy completed, indicating a high level of completeness of the genome.

**Conclusion:**

The platinum-grade genome assembly and its annotation provide valuable information for future in-depth comparative genomics studies, including sexual determination analysis in *T. rubrofasciata* and the pathogenesis of Chagas disease.

## Data Description

### Introduction

The insect *Triatoma rubrofasciata* (De Geer) (Hemiptera, Triatominae) is the first Triatominae species formally described, initially with the name *Cimex rubrofasciatus* De Geer, 1773 [1]. This insect presents anthropogenic habits with its dispersion favored by the interaction between residential settlement and human activities [2]. It is considered of global epidemiological importance because it has a pantropical widespread distribution that is found in approximately 45 countries from the Old World to the New World [3]. It is one of the 151 species of Triatominae that has 18 genera currently described worldwide that can transmit American trypanosomiasis, known as Chagas disease [4]. This condition has a great impact on public health, with 7–8 million people estimated to be infected worldwide, mostly in Latin America. It has become a global health issue in this century with the spread to nonendemic countries due to growing population movements [5].

Due to growing population movements, important epidemiological changes have occurred in recent decades, and the disease has now spread to many nonendemic countries [6]. The widespread of *T. rubrofasciata* emerges as a potential risk of outbreaks in these regions, which demands urgent studies through comprehensive sampling and comparative studies. The lack of a high-quality reference genome represents a major hurdle for such efforts. Here, we present a platinum-grade reference genome for *T. rubrofasciata*, which will be valuable for developing vector control programs.

### Sample description and DNA sequencing

**Figure 1:**
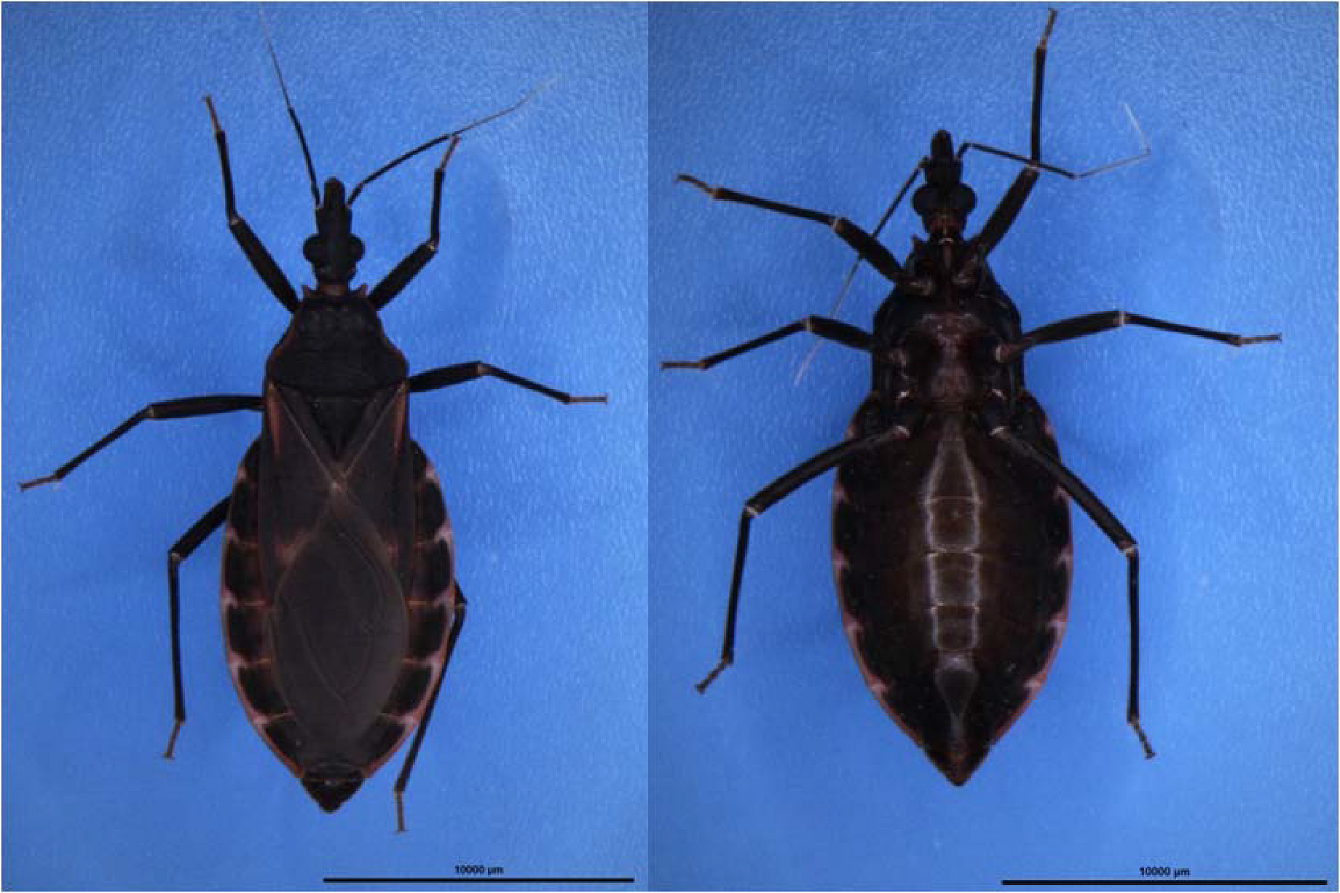
Dorsal (left) and ventral (right) views of a female *Triatoma rubrofasciata*.

An adult female insect *T. rubrofasciata* (Fig. 1) was used for reference genome construction in this study. This insect was the second-generation offspring of a population that was established from the eggs of single female adult collected in Shunde County, Foshan City, Guangdong Province (22°42′44.63″N, 113°08′45.34″E), China, in 2016 [7]. DNA was extracted from this individual using the traditional phenol/chloroform extraction method and was quality checked using agarose gel electrophoresis. A single band was observed, indicating high integrity of DNA molecules for library construction for the Illumina X Ten (Illumina, Inc., San Diego, CA, USA) and the PacBio Sequel (Pacific Biosciences of California, Menlo Park, CA, USA) sequencing platforms.

**Figure 2:**
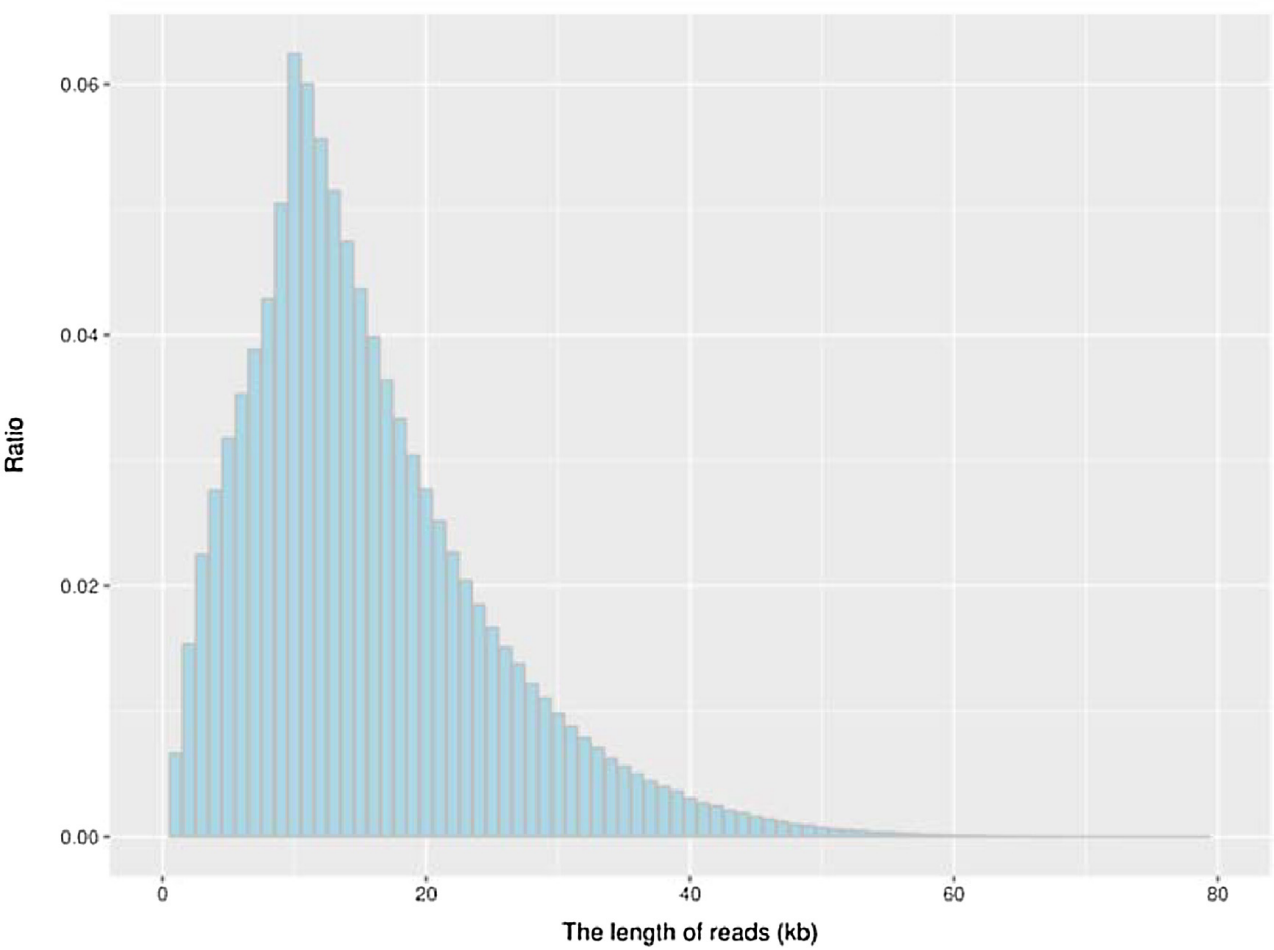
The plot of the read length distribution/ratio of the subreads.

Using DNA preparation, a library with an insertion length of 350 bp was constructed for the Illumina sequencing platform according to the manufacturer's protocol. In total, 46.75-Gb short reads were obtained from the Illumina X Ten DNA sequencing platform (Table 1), and 39.32-Gb filtered reads were used for the following genome survey analysis and for final-stage base-level genome sequence polishing. Meanwhile, 20-kb libraries were constructed for PacBio Sequel sequencing. Using 14 SMRT cells, 8.23 million reads were generated, with the total length of 69.38 Gb (Table 1). The mean length of these subreads was 8.43 kb, and the plot of the read length distribution/ratio is shown in Fig. 2.

**Table 1:** Sequencing data generated for *Triatoma rubrofasciata* genome assembly and annotation

Library type	Platform	Library size (bp)	Data size (Gb)	Application
Short reads	HiSeq X Ten	350	46.75	Genome survey and genomic base correction
Long reads	PacBio Sequel	20,000	69.38	Genome assembly
Hi-C	HiSeq X Ten	300–500	103.61	Chromosome construction

### Genome features estimation through k-mer analysis

With sequencing data from the Illumina HiSeq X Ten DNA sequencing platform, several genome features were evaluated for the genome of *T. rubrofasciata*. To ensure the quality of the analysis, ambiguous bases and low-quality reads were first trimmed and filtered using the HTQC package [8]. First, the quality of bases at 2 read ends was checked. Bases in sliding 5-bp windows were deleted if the average quality of the window was below 20. Second, reads were filtered if the average quality was smaller than 20 or the read length was shorter than 75 bp. Third, the mate reads were also removed if the corresponding reads were filtered.

**Figure 3:**
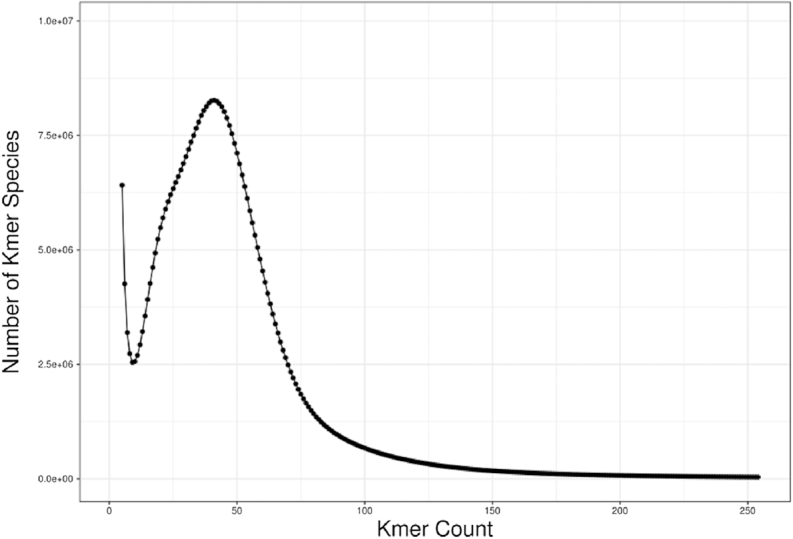
17-mer depth distribution for genome size estimation analysis of *Triatoma rubrofasciata*.

The processed reads were used for genome assessment. We calculated the number of each 17-mer from the sequencing data using the jellyfish software (v2.1.3) [9], and the distribution was analyzed with GCE software [10]. We estimated the genome size of 757 Mb with the heterozygosity of 1.01% and repeat content of 55.49% in the genome. K-mer analysis was used to estimate the genome size, which showed the PacBio assembly was of good quality (Fig. 3). The genome size of *T. rubrofasciata* is similar to that of *Rhodnius prolixus*, another insect vector of Chagas disease, which has a predicted 733-Mb genome size [11].

### Genome assembly using PacBio long reads

FALCON [12] was employed using the length_cut_off and length_cutoff_pr parameters of 3 kb and 3 kb, respectively. We first obtained a 677.72-Mb genome with a contig N50 of 2.71 Mb. The genome sequences were subsequently polished using PacBio long reads using arrow [13] and Illumina short reads by pilon [14] to correct base errors.

### *In situ* Hi-C library construction and chromosome assembly using Hi-C data

A separate female individual *T. rubrofasciata* was used for library construction for Hi-C analysis as described previously [15, 16]. The library was sequenced with a 150-bp paired-end mode on the Illumina HiSeq X Ten platform.

From the Illumina HiSeq X Ten platform, 103.61-Gb reads were obtained for the Hi-C library and 99.28-Gb filtered reads were used for the following Hi-C analysis. The reads were mapped to the above *T. rubrofasciata* genome with Bowtie [17], with both ends of paired reads being mapped to the genome separately. To increase the interactive Hi-C reads ratio, an iterative mapping strategy was performed as in previous studies, and only read pairs that both ends uniquely mapped were used for the following analysis. From the alignment of the paired ends, self-ligation, nonligation, and other sorts of invalid reads, including StartNearRsite, PCR amplification, random break, LargeSmallFragments, and ExtremeFragments, were filtered out by Hi-C library, with the method described in a previous study [15]. Through the recognition of restriction sites in sequences, contact counts among contigs were calculated and normalized.

**Figure 4:**
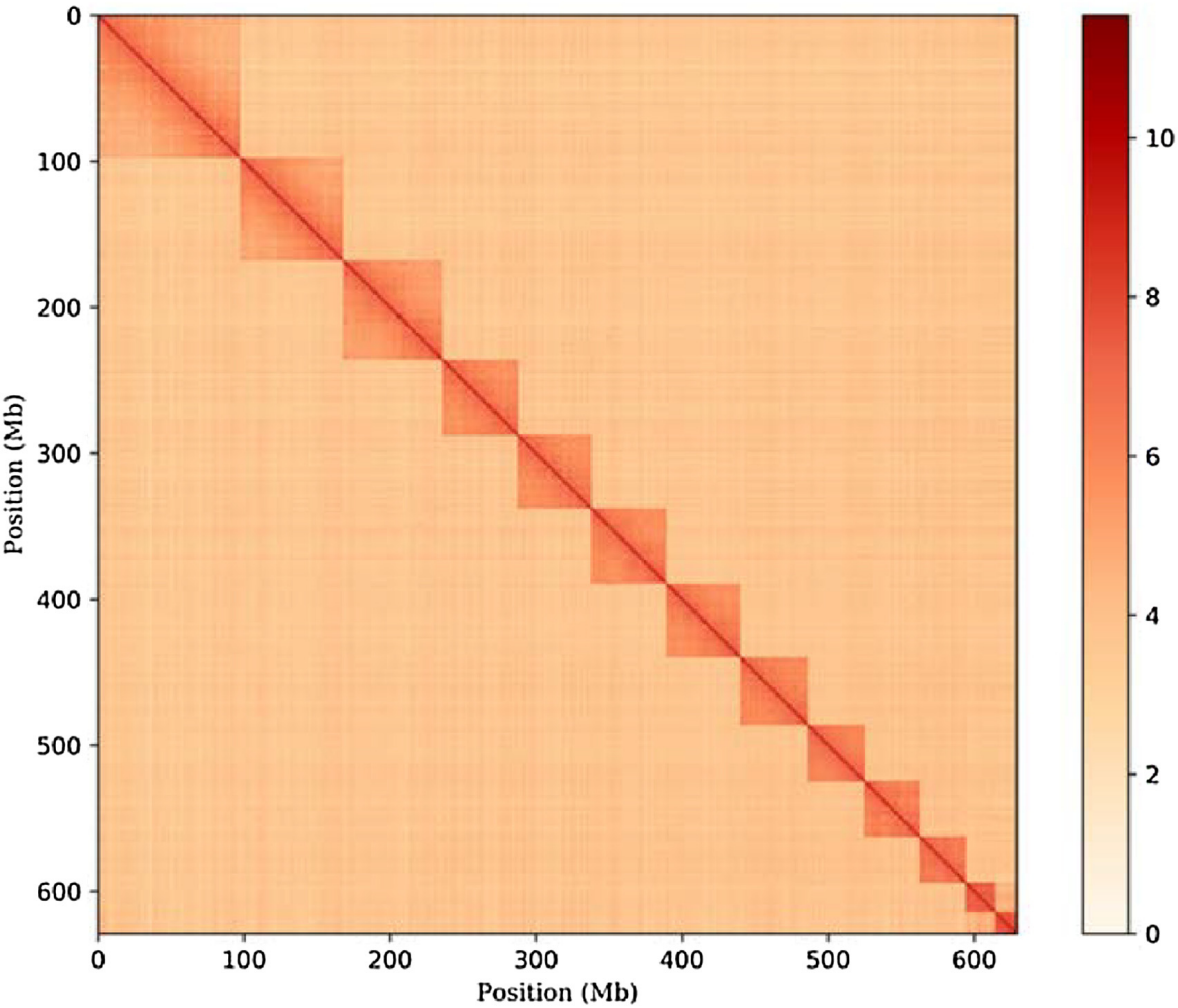
DNA interaction heatmap generated in Hi-C analysis (resolution: 500 Kb).

By clustering the contigs using the contig contact frequency matrix, we were able to correct some minor errors in the FALCON assembly results. Contigs with errors were corrected by breaking into shorter contigs, and we obtained a chromosome-level genome assembly of 680.73 Mb with 2126 contigs and a contig N50 of 2.72 Mb. The longest contig was 10.27 Mb in size (Table 2). Among these 2126 contigs, 626 contigs were mounted to 13 chromosomes with Lachesis [18] using the agglomerative hierarchical clustering method. Lachesis was further applied to order and orient the clustered contigs according to the contact matrix. Contigs anchored to chromosomes accounted for 92.51% of the total genome bases (Fig. 4). The number of chromosomes matched nicely to a previously published karyotype of a female *T. rubrofasciata* individual (2n = 24 = 11 * 2 + X1 + X2) [1]. Taken together, we have successfully reconstructed the first chromosomal-level assembly of *T. rubrofasciata* of 680.73 Mb, with 2126 contigs, a contig N50 of 2.72 Mb, and a scaffold N50 of 50.70 Mb (Table 2).

**Table 2:** Statistics for genome assembly of *Triatoma rubrofasciata*

Sample	Length		Number	
	Contig (bp)	Scaffold (bp)	Contig	Scaffold
Total	680,314,598	680,726,098	2126	1303
Max	10,270,547	97,329,580	–	–
N50	2,722,109	50,700,875	76	6
N60	2,121,675	50,415,845	104	7
N70	1,587,961	46,556,423	140	8
N80	1,038,484	37,928,883	193	10
N90	338,786	20,341,594	301	12

### Genome quality evaluation

We assessed the quality of genome of *T. rubrofasciata* in 3 aspects: sequence continuity, genome completeness, and base-level accuracy.

**Figure 5:**
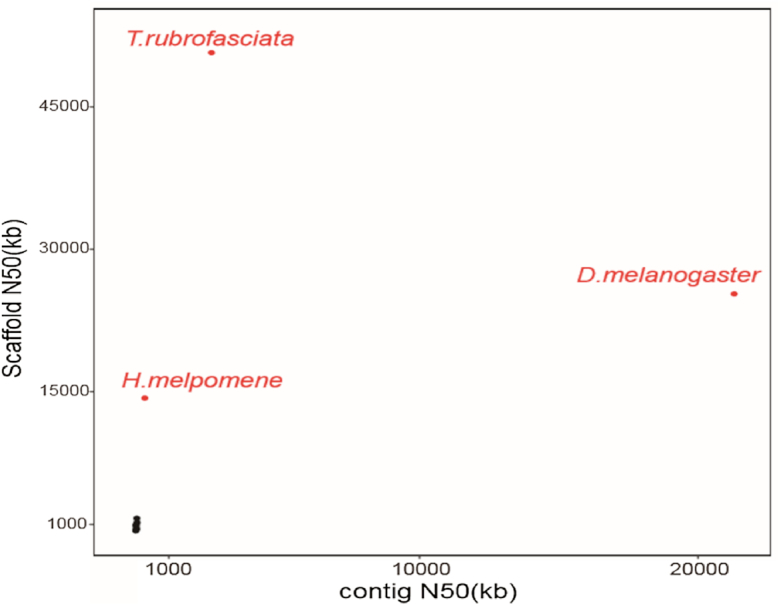
Genome assembly comparison of *Triatoma rubrofasciata* with other sequenced insect genomes (*Apis mellifera, Acyrthosiphon pisum, Cimex lectularius, Culex quinquefasciatus, Drosophila melanogaster, Gerris buenoi, Glossina palpalis, Halyomorpha halys, Heliconius melpomene, Homalodisca vitripennis, Oncopeltus fasciatus, Rhodnius prolixus*). The *x*- and *y*-axes represent the contig and scaffold N50s, respectively. The genomes with both contig and scaffold N50s less than 2M are hignlighted in black.

First, we compared the contig/scaffold number and N50 length of contig of *T. rubrofasciata* with insect species with sequenced genomes and found that our assembly has much improved quality over other insects (Fig. 5). We attributed the improvement to the application of the PacBio long reads for genome assembly. With Hi-C data analysis, we successfully assembled the *T. rubrofasciata* genome to chromosome level with just 1 individual. Like previous studies, insect genome heterozygosity was one of the biggest challenges for genome assembly, both in terms of contig and scaffold assembly. Traditional chromosomal genome assembly requires physical maps and genetic maps, which is enormously time- and labor-consuming. Our work illustrated that the genome assembly using PacBio long sequencing data was not only affordable but also effective for overcoming the difficulties presented by insect genome assembly.

Second, the assembled genome was subjected to the BUSCO v.3.0.2 (RRID:SCR_015008) [19] to assess the completeness of the genome assembly. We used the “insect_obd9” gene set, and 98.2% of the BUSCO genes were identified in the*T. rubrofasciata* genome. More than 95.1% of BUSCO genes were single-copy completed in our genome, illuminating a high level of completeness of the genome.

Third, NGS short reads were aligned to the genome using BWA [20]. About 98.1% of reads were aligned to the genome, of which 98.0% were reads paired aligned. The insertion length distribution of read pairs exhibited a single peak around 300 bp, which was consistent with the design for the Illumina sequencing library construction. Note that the NGS data, which were used for error correction, were not used in the contig assembly. Therefore, the insertion length distribution of NGS data illustrated the high quality of our assembly at the contig level. From the NGS reads alignment, we detected 8478 homologous SNP loci using GATK [21], demonstrating the high base-level accuracy of 99.99%.

### Repeat element and gene annotation

TRF [22] was used for repetitive element identification in the*T. rubrofasciata* genome. A *de novo* method applying RepeatModuler [23] was used to detect TEs. The resulting *de novo* data, combined with a known repeat library from Repbase [24], were used to identify TEs in the *T. rubrofasciata* genome by RepeatMasker [25].

**Figure 6:**
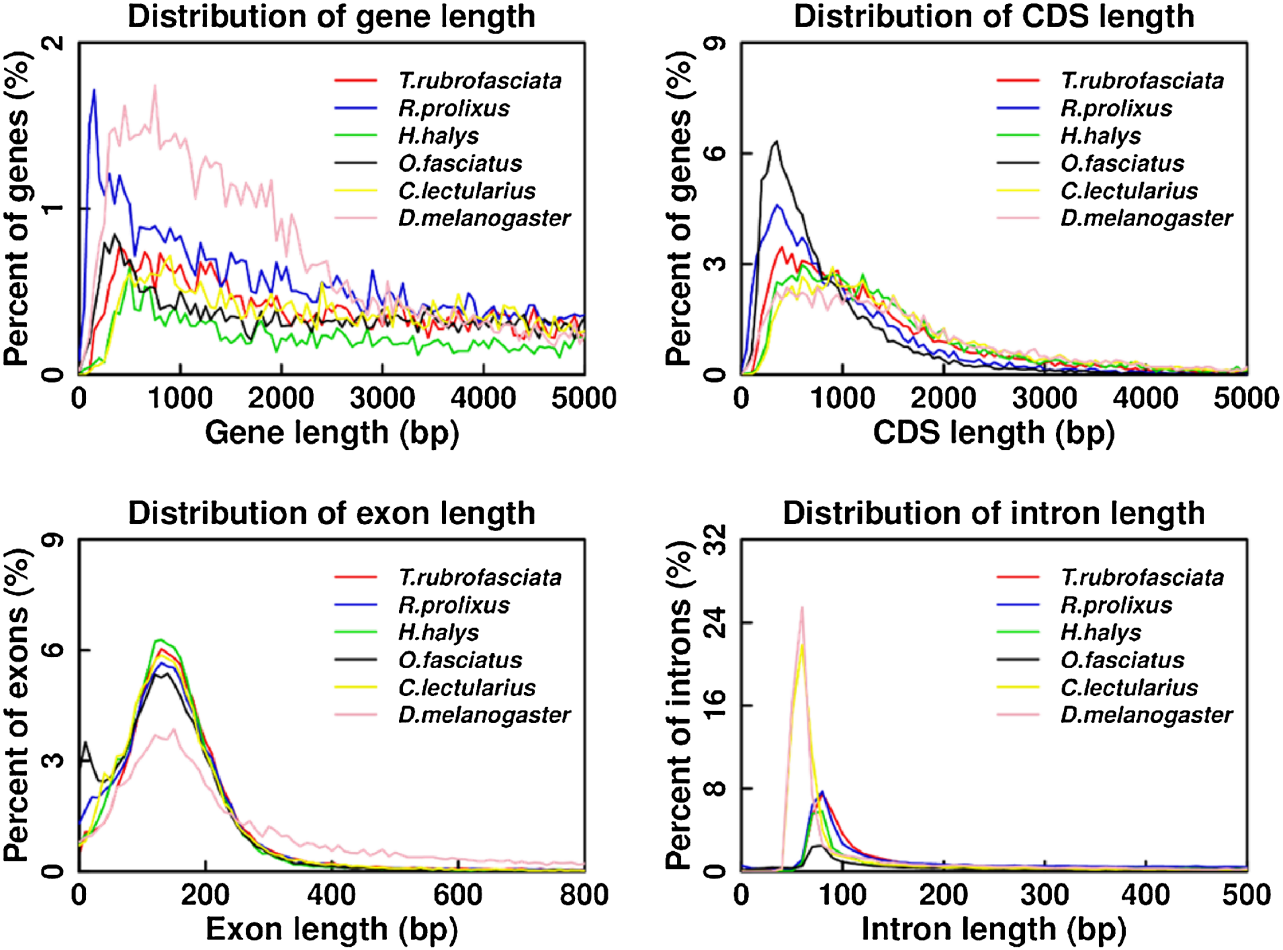
Length distribution comparison on total gene, CDS, exon, and intron of annotated gene models of *Triatoma rubrofasciata* with other closely related insect species. Length distribution of total gene (A), CDS (B), exon (C), and intron (D) was compared to those of *Rhodnius prolixus, Halyomorpha halys, Oncopeltus fasciatus, Cimex lectularius*, and *Drosophila melanogaster*.

Protein-coding genes in the *T. rubrofasciata* genome were annotated using the *de novo* program Augustus (RRID:SCR_008417) [26]. Protein sequences of the closely related species, including *Rhodnius prolixus* (from VectorBase), *Halyomorpha halys* (from NCBI), *Oncopeltus fasciatus* (from USDA), *Cimex lectularius* (from NCBI), and *Drosophila melanogaster* (from NCBI), were aligned to the *T. rubrofasciata* genome with tblastn. Full-length transcripts obtained using Iso-Seq were mapped to the genome using Gmap [27]. Finally, gene models predicted from all above methods were combined by MAKER [28], resulting in 12,691 protein-coding genes. The gene number, gene length, CDS length, exon length, and intron length distribution were all comparable with the related insects (Fig. 6).

To functionally annotate protein-coding genes in the *T. rubrofasciata* genome, we searched all predicted gene sequences to NCBI nonredundant protein (NR), InterPro (InterProScan, RRID:SCR_005829) [29], GO, KEGG (RRID:SCR_012773) [30], Swissprot [31], and TrEMBL databases [31] by BLASTN [32] and BLASTX [33]. A threshold e-value of 1e-5 was used for all BLAST applications. Finally, 12,063 genes were functionally annotated (Table 3).

**Table 3:** Statistics for genome annotation of *Triatoma rubrofasciata*

Database	Number	Percent
NR	11,451	90.23
InterPro	9625	75.84
GO	7180	56.58
KEGG ALL	10,867	85.63
KEGG KO	6112	48.16
Swissprot	9448	74.45
TrEMBL	11,989	94.47
Total	12,063	95.05

### Phylogenetic analysis of *T. rubrofasciata* with other insects

OrthMCL was used to cluster gene families. First, proteins from *T. rubrofasciata* and the closely related insects, including *Rhodnius prolixus, Oncopeltus fasciatus, Halyomorpha halys, Cimex lectularius, Drosophila melanogaster, Gerris buenoi, Homalodisca vitripennis, Acyrthosiphon pisum, Culex quinquefasciatus, Glossina palpalis, Apis mellifera*, and *Heliconius melpomene*, were all-to-all blasted by BLASTP [33] utility with an e-value threshold of 1e-5. Only proteins from the longest transcript were used for genes with alternative splices. We identified 21,850 gene families for *T. rubrofasciata* and the related species, among them 330 single-copy ortholog families.

**Figure 7:**
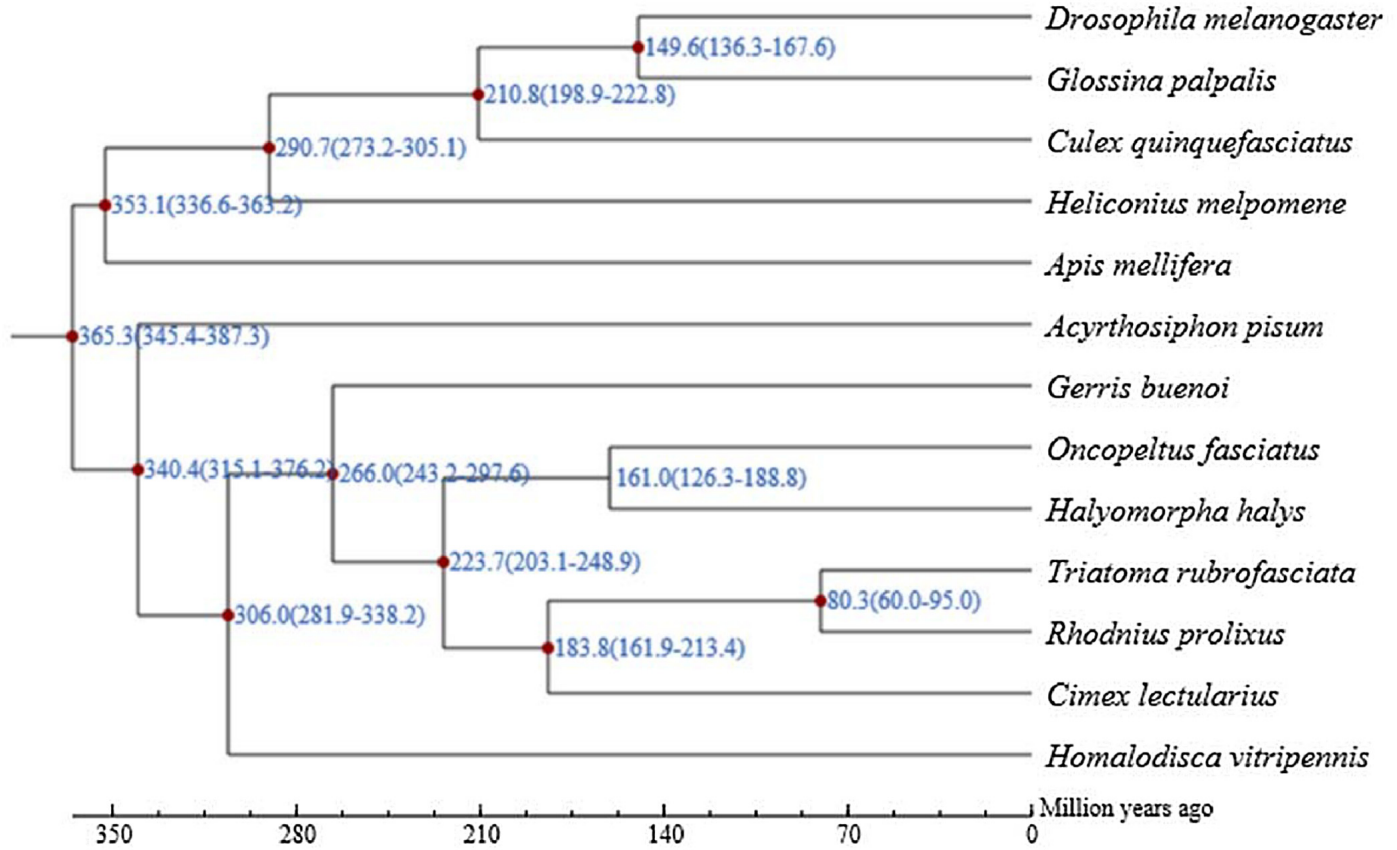
Phylogenetic analysis of *T. rubrofasciata* with other insect species. The estimated species divergence time (million years ago) and the 95% confidential intervals are labeled at each branch site. The divergence used for time recalibration is illuminated as red dots in the tree.

Using single-copy orthologs, we probed the phylogenetic relationships for *T. rubrofasciata* and other insects. To this end, protein sequences of single-copy genes were aligned using MUSCLE [34]. Guided by the protein multisequence alignment, the alignment of the CDS for those genes was generated and concatenated for the following analysis. The phylogenetic relationships were constructed using PhyML [35] using the concatenated nucleotide alignment with the JTT+G+F model. We first obtained divergent times for all pairs with the phylogenetic tree using r8s [36], which were used as input, together with molecular clock data from the divergence time from the TimeTree database [37], to estimate species divergence time for all pairs of species in the phylogenetic tree using the MCMCtree program (from PAML) [38]. We found that *T. rubrofasciata* was most closely related to *R. prolixus*, and the 2 species diverged from their common ancestor around 60.00–95.00 MYA (Fig. 7).

## Conclusion

We reconstructed the first high-quality, chromosome-level assembly of *T. rubrofasciata* using an integrated strategy of PacBio, Illumina, and Hi-C technologies. Using the long reads from the PacBio Sequel platform and short reads from the Illumina HiSeq X Ten platform, we successfully constructed contig assembly for *Triatoma*. Leveraging contact information among contigs from Hi-C technology, we further improved the assembly to the chromosome-level quality. We annotated 12,691 protein-coding genes in the *T. rubrofasciata* genome, 12,063 of which were functionally annotated. With 330 single-copy orthologs from *T. rubrofasciata* and other related insects, we constructed the phylogenetic relationship of these insects and found that *T. rubrofasciata* might have diverged from its common ancestor of *R. prolixus* around 60.00–95.00 MYA. Given the increasing interests in insect genome evolution and the biological importance of *T. rubrofasciata* as the vector for Chagas disease, our genomic and transcriptome data provide a valuable genetic resource for the following functional genomics investigations for the research community.

## Availability of supporting data

The raw data from our genome project was deposited in the NCBI Sequence database with Bioproject IDs PRJNA516044. The Illumina, PacBio, and Hi-C sequencing data are available from NCBI via accession numbers SRR8466736, SRR8466737, and SRR8466756, respectively. The Illumina transcriptome sequencing data were deposited to NCBI via accession numbers SRR8468315 and SRR8468316. Other data further supporting this work are available in the *GigaScience*repository, GigaDB [39].

## Ethics Statement

This study was approved by the Animal Care and Use Committee of the National Institute of Parasitic Diseases, Chinese Center for Disease Control and Prevention. All participants consented to the study under the "Ethics, Consent and Permissions" heading. All participants consented to publish the work under the "Consent to Publish" heading.

## Abbreviations

BLAST, Basic Local Alignment Search Tool; bp, base pairs; BUSCO, Benchmarking Universal Single-Copy Orthologs; CDS, coding DNA sequences; Gb, gigabase pairs; GO, Gene Ontology; Hi-C, High-throughput/resolution chromosome conformation capture; kb, kilobase pairs; KEGG, Kyoto Encyclopedia of Genes and Genomes; Mb, megabase pairs; MYA, million years ago; NCBI, National Center for Biotechnology Information; NGS, Next Generation Sequencing; NR, nonredundant protein; PCR, polymerase chain reaction; SMRT, Single Molecule Real Time; SNP, single-nucleotide polymorphism; TE, transposable element; TRF, Tandem Repeat Finder; USDA, US Department of Agriculture.

## Competing interests

The authors declare that they have no competing interests.

## Funding

This work was supported by the National Key Research and Development Program of China (Grant No. 2016YFC1202000), the National Science and Technology Project (No. 2018ZX10101002), and the CAS Pioneer Hundred Talents Program (to N.S.C.) and Taishan Scholar Project Special Fund (to N.S.C.).

## Author Contributions

Z.X.N., L.Q., Z.Y., and H.W. conceived the project. L.Q., G.Y.H., Z.Y., Z.D., L.Y.Y., W.J.T., and Z.Z.B. collected the samples and extracted the DNA and RNA. L.Q, G.Y.H., and Z.Y. performed the genome assembly and data analysis. C.N.S. performed the data analysis. L.Q. and C.N.S. wrote the paper. Z.X.N. revised the manuscript. All authors read, edited, and approved the final version of the manuscript.

## Supplementary Material

giz089_GIGA-D-19-00028_Original_Submission

giz089_GIGA-D-19-00028_Revision_1

giz089_GIGA-D-19-00028_Revision_2

giz089_Response_to_Reviewer_Comments_Original_Submission

giz089_Response_to_Reviewer_Comments_Revision_1

giz089_Reviewer_1_Report_Original_Submission Ben Mans -- 2/25/2019 Reviewed

giz089_Reviewer_1_Report_Revision_1 Ben Mans -- 5/13/2019 Reviewed

giz089_Reviewer_2_Report_Original_Submission Ting-Fung Chan, PhD -- 3/9/2019 Reviewed

giz089_Reviewer_2_Report_Revision_1 Ting-Fung Chan, PhD -- 5/18/2019 Reviewed

giz089_Supplemental_File
