# Supplementary material for: A chromosomal-level genome assembly for the insect vector for Chagas disease, Triatoma rubrofasciata
Source: Gigascience. 2019 Aug 19;8(8):giz089. doi: 10.1093/gigascience/giz089 (PMC6699579; doi:10.1093/gigascience/giz089)

GigaScience

A Chromosomal-Level Genome Assembly for the insect vector for Chagas disease, Triatoma rubrofasciata

--Manuscript Draft--

Manuscript Number:	GIGA-D-19-00028
Full Title:	A Chromosomal-Level Genome Assembly for the insect vector for Chagas disease, Triatoma rubrofasciata
Article Type:	Data Note
Funding Information:	Foundation for the Development of Science and Technology Museums in China (Grant No. 2016YFC1202000) — Prof. Xiao-Nong Zhou
Abstract:	Background<br><br>Triatoma rubrofasciata is a widespread pathogen vector for Chagas disease, an illness that affects approximately seven million people worldwide. Despite of its importance to human health, its evolutionary origin has not been conclusively determined. A reference genome for T. rubrofasciata is not yet available.<br><br>Finding<br>We have sequenced the genome of a female T. rubrofasciata individual using a single molecular DNA sequencing technology (i.e., PacBio DNA sequencing) and have successfully reconstructed a whole-genome assembly that covers 99% of the nuclear genome (~677 Mb). Through Hi-C analysis, we have reconstructed full-length chromosomes of this female individual that has 13 unique chromosomes (2n = 24 = 22 + X1 + X2) with a contig N50 of 2.96 Mb and a scaffold N50 of 51.38 Mb. This genome has high base-level accuracy of 99.99%. This platinum-grade genome assembly has 12,695 annotated protein-coding genes. More than 97% BUSCO gene were single-copy completed, indicating a high level of completeness of the genome.<br><br>Conclusion<br>The platinum-grade genome assembly and its annotation provide valuable information for future in-depth comparative genomics studies including sexual determination analysis in T. rubrofasciata and the pathogenesis of Chagas disease.<br><br>Key Words: Triatoma rubrofasciata, PacBio DNA sequencing, Hi-C, chromosomal-level assembly, comparative genomics, RNA-Seq, Iso-Seq
Corresponding Author:	Xiao-Nong Zhou<br>National Institute of Parasitic Diseases<br>Shanghai, CHINA
Corresponding Author Secondary Information:	
Corresponding Author's Institution:	National Institute of Parasitic Diseases
Corresponding Author's Secondary Institution:	
First Author:	Qin Liu, Ph.D.
First Author Secondary Information:	
Order of Authors:	Qin Liu, Ph.D.
	Yunhai Guo
	Yi Zhang
	Wei Hu
	Yuanyuan Li
	Dan Zhu

Powered by Editorial Manager® and ProduXion Manager® from Aries Systems Corporation

	Zhengbin Zhou
	Jiatong Wu
	Lansheng Chen
	Xiao-Nong Zhou
**Order of Authors Secondary Information:**	
**Additional Information:**	
**Question**	**Response**
Are you submitting this manuscript to a special series or article collection?	No
**Experimental design and statistics**<br><br>Full details of the experimental design and statistical methods used should be given in the Methods section, as detailed in our Minimum Standards Reporting Checklist. Information essential to interpreting the data presented should be made available in the figure legends.<br><br>Have you included all the information requested in your manuscript?	Yes
**Resources**<br><br>A description of all resources used, including antibodies, cell lines, animals and software tools, with enough information to allow them to be uniquely identified, should be included in the Methods section. Authors are strongly encouraged to cite Research Resource Identifiers (RRIDs) for antibodies, model organisms and tools, where possible.<br><br>Have you included the information requested as detailed in our Minimum Standards Reporting Checklist?	Yes
**Availability of data and materials**<br><br>All datasets and code on which the conclusions of the paper rely must be either included in your submission or	Yes

deposited in publicly available repositories (where available and ethically appropriate), referencing such data using a unique identifier in the references and in the "Availability of Data and Materials" section of your manuscript.<br><br>Have you have met the above requirement as detailed in our Minimum Standards Reporting Checklist?	

Powered by Editorial Manager® and ProduXion Manager® from Aries Systems Corporation

Click here to download Manuscript Triatoma rubrofasciata genome.docx

A Chromosomal-Level Genome Assembly for the insect vector for Chagas disease, *Triatoma rubrofasciata*

Qin Liu[1#], Yunhai Guo[1#], Yi Zhang[1#], Wei Hu[1,2] ,Yuanyuan Li[1], Dan Zhu[1], Zhengbin Zhou[1], Jiatong Wu[1], Lansheng Chen[3,4,5*], Xiao-Nong Zhou[1*]

[1]National Institute of Parasitic Diseases, Chinese Center for Disease Control and Prevention; Key Laboratory of Parasite and Vector Biology, Ministry of Health; WHO Collaborating Center for Tropical Diseases, Shanghai 200025, P. R. China

[2]Department of Microbiology and Microbial Engineering，School of Life Sciences，Fudan, Shanghai 200025, P. R. China

[3]CAS Key laboratory of Marine Ecology and Environmental Sciences, Institute of Oceanology, Chinese Academy of Sciences; Qingdao, Shandong 266071, China；

[4]Laboratory for Marine Ecology and Environmental Science, Qingdao National Laboratory for Marine Science and Technology, Qingdao, Shandong 266237, China；

[5]Department of Molecular Biology and Biochemistry, Simon Fraser University, Burnaby, Canada

Abstract

Background:

Triatoma rubrofasciata is a widespread pathogen vector for Chagas disease, an illness that affects approximately seven million people worldwide. Despite of its importance to human health, its evolutionary origin has not been conclusively determined. A reference genome for *T. rubrofasciata* is not yet available.

Finding:

We have sequenced the genome of a female *T. rubrofasciata* individual using a single molecular DNA sequencing technology (i.e., PacBio DNA sequencing) and have successfully reconstructed a whole-genome assembly that covers 99% of the nuclear genome (~677 Mb). Through Hi-C analysis, we have reconstructed full-length chromosomes of this female individual that has 13 unique chromosomes (2n = 24 = 22 + X1 + X2) with a contig N50 of 2.96 Mb and a scaffold N50 of 51.38 Mb. This genome has high base-level accuracy of 99.99%. This platinum-grade genome assembly has 12,695 annotated protein-coding genes. More than 97% BUSCO gene were single-copy completed, indicating a high level of completeness of the genome.

Conclusion:

The platinum-grade genome assembly and its annotation provide valuable information for future in-depth comparative genomics studies including sexual determination analysis in *T. rubrofasciata* and the pathogenesis of Chagas disease.

Key Words: *Triatoma rubrofasciata*, PacBio DNA sequencing, Hi-C, chromosomal-level assembly, comparative genomics, RNA-Seq, Iso-Seq

Data description

Introduction

T. rubrofasciata (De Geer) (Hemiptera, Triatominae) is the first Triatominae species formally described, as *Cimex rubrofasciatus* De Geer, 1773 [1]. This insect presents anthropogenic habits with its dispersion favored by the interaction between residential settlement and human activities [2]. It is considered of global epidemiological importance since it has a pantropical widespread distribution which is found in approximately 45 countries from the Old World to the New World [3]. It is one of the 151 species of Triatominae that has 18 genera currently described worldwide that can transmit American trypanosomiasis known as Chagas disease [4]. This condition has great impact on public health, with 7-8 million people estimated to be infected worldwide, mostly in Latin America. It has become a global health issue in this century with the spread to the non-endemic countries due to growing population movements [5].

Due to growing population movements, important epidemiological changes have occurred in recent decades, and the disease has now spread to non-endemic countries [6] The widespread of *T. rubrofasciata* emerges as a potential risk of outbreaks in these regions, which demand urgent studies through comprehensive sampling and comparative studies. The lack of a high-quality reference genome represents a major hurdle for such efforts. Here, we present our effort in reconstructing a platinum-grade reference genome for *T. rubrofasciata*, which will be valuable for developing vector control programs.

Sample preparation and DNA sequencing

An adult female insect (Figure 1) was used for reference genome construction in this study. This insect was the second generation offspring of the population which was established from the eggs of single female adult collected in Shunde County, Foshan City, Guangdong Province (22°42′44.63″N, 113°08′45.34″E), China, in 2016 [7]. DNA was extracted from this individual using the traditional phenol/chloroform extraction method and was quality checked using agarose gel electrophoresis. A single band was observed, indicating the integrity of DNA molecules for library construction for the Illumina X Ten (Illumina Inc., San Diego, CA, USA) and the PacBio Sequel (Pacific Biosciences of California, Menlo Park, CA, USA) sequencing platforms.

Using the DNA preparation, a library with the insertion length of 300 bp was constructed for Illumina sequencing platform according to the manufacturer’s protocol. 53.7 Gb short reads were obtained from the Illumina X Ten sequencing (Table 1). 49.7 Gb filtered reads were used for the following genome survey analysis, and for final-stage base-level genome sequence correction. Meanwhile, two 20 Kb-libraries were constructed for PacBio Sequel sequencing. Using two sequencing SMRT cells, 8.23 million reads were generated, with the total length of 69.38 Gb (Table 1). The mean length of these polymerases was 8.43 Kb.

Genome features estimation through *K*mer analysis

With sequencing data from the Illumina platform, several genome features were evaluated for the genome of *T. rubrofasciata*. To ensure the quality of the analysis, ambiguous bases and low-quality reads were first trimmed and filtered using the HTQC package [8]. The following quality control was performed under the framework of HTQC. First, the quality of bases at two read ends was checked. Bases in sliding 5 bp windows were deleted if the average quality of the window was below 20. Second, reads were filtered if the average quality were smaller than 20 or the read length was shorter than 75 bp. Third, the mate reads were also removed if the corresponding reads were filtered.

The processed reads were used for genome assessment. We calculated the number of each 17-mer from the sequencing data using the jellyfish software (v2.1.3) [9], and the distribution was analyzed with GCE software. We estimated the genome size of 720 Mb with the heterozygosity of 1.02% and repeat content of 52.43% in the genome. The genome size of *T. rubrofasciata* is similar to that of *Rhodnius prolixus*, another insect vector of Chagas disease, which has a predicted 733 Mb genome size [10].

Genome assembly using PacBio long reads

FALCON [11] was employed using the length_cutoff and pr_length_cutoff parameters of 3 Kb and 3 Kb, respectively. We obtained 677.72 Mb genome with 2115 contigs, with a contig N50 of 2.71 Mb (Table 2). The longest contig was 10.22 Mb in size. The genome sequences were subsequently polished by PacBio long reads using arrow [12] and Illumina short reads by pilon [13] to correct base errors. The corrected genome was further applied for the following chromosome assembly construction using Hi-C data.

***In situ* Hi-C library construction and chromosome assembly using Hi-C data**

A separate individual female *T. rubrofasciata* was used for library construction for Hi-C analysis as described previously [14, 15]. Finally, the library was sequenced with 150 paired-end mode on the Illumina HiSeq X Ten platform (San Diego, CA, United States).

From the Illumina sequencing platform, 683.26 million paired-end reads were obtained for the Hi-C library. The reads were mapped to the above *T. rubrofasciata* genome with Bowtie [16], with two ends of paired reads being mapped to the genome separately. To increase the interactive Hi-C reads ratio, an iterative mapping strategy was performed as previous studies, and only read pairs that both ends uniquely mapped were used for the following analysis. From the alignment status of two ends, self-ligation, non-ligation and other sorts of invalid reads, including StartNearRsite, PCR amplification, random break, LargeSmallFragments and ExtremeFragments, were filtered out by Hi-Clib and the method was described in a previous study [14]. Through the

recognition of restriction sites in sequences, contact counts among contigs were calculated and normalized.

According to previous karyotype analyses, the genome of a female *Triatoma rubrofasciata* individual has 13 (2n = 24 = 11 * 2 + X1 + X2) chromosomes [1]. By clustering the contigs using the contig contact frequency matrix, we were able to correct some minor errors in the FALCON assembly results. Contigs with errors were corrected by broking into shorter contigs, and many contigs were merged to form longer contigs. We obtained a chromosome-level genome assembly with 626 contigs, substantially fewer than the 2115 contigs in the FALCON assembly. We successfully organized these contigs into 13 groups in Lachesis [17] using the agglomerative hierarchical clustering method. Lachesis was further applied to order and orient the clustered contigs according to the contact matrix. As a result, 626 contigs were reliably anchored, ordered and orientated on chromosomes, accounting for 96.25% of the total genome bases (Figure 2). Then, we applied PBJelly [18] to fill the gaps and to merge the contig sequences using PacBio long reads. Finally, the first chromosomal-level assembly of *T. rubrofasciata* with 626 contigs, a contig N50 of 2.96 Mb and a scaffold N50 of 51.38 Mb was constructed.

Genome quality evaluation

We assessed the quality of genome of *T. rubrofasciata* after the assembly process. The quality evaluation was carried out in three aspects: continuity, completeness and base level accuracy.

First of all, we compared the sequence number and N50 length of contig of *T. rubrofasciata* with insect species with sequenced genomes and found that our assembly has much improved quality over other insects (Figure 3). We attributed the advantage to the application of the PacBio long reads for genome assembly. As previous studies, genomic heterozygosity of insects was one of the biggest challenges for genome assembly, both in terms of contig and scaffold assembly. Our work illustrated that the genome assembly using PacBio long sequencing data was not only affordable but also effective for overcoming the difficulty of mollusk genome assembly. Traditional chromosomal genome assembly requires physical maps and genetic maps, which is enormously time- and labor-consuming. With Hi-C data analysis, we successfully assembled *T. rubrofasciata* genome into chromosome-level with just one individual.

Second, the assembled genome was subjected to the BUSCO (version 3.0) [19] to assess the completeness of the genome. 98% of the BUSCO genes were identified in *T. rubrofasciata* genome. More than 97% BUSCO gene were single-copy completed in our genome, illuminating a high level of completeness of the genome.

Third, NGS short reads were aligned to the genome using BWA [20]. About 98.1% of reads were aligned to the genome, of which 97.39% were reads paired aligned. The insertion length distribution of read pairs exhibited a single peak around 300 bp, which was consistent with the design for the Illumina sequencing library construction. The NGS data, which was used for error

correction, was not used in contig assembly. Therefore, the insertion length distribution of NGS data illustrated the high quality of our assembly at the contig level. From the NGS reads alignment, we detected 8934 homologous SNP loci using GATK [21], demonstrating the high base-level accuracy of 99.99%.

Repeat element and gene annotation

Tandem Repeat Finder (TRF) [22] was used for repetitive element identification in *T. rubrofasciata* genome. A *de novo* method applying RepeatModuler (http://www.repeatmasker.org/RepeatModeler.html) was used to detect transposable elements (TEs). The resulted *de novo* data, combined with known repeat library from Repbase [23], were used to identify TEs in the *T. rubrofasciata* genome by RepeatMasker [24].

Protein-coding genes in the *T. rubrofasciata* genome were annotated using the *de novo* program Augustus (RRID:SCR_008417) [25]. Protein sequences of the closely related species including *Rhodnius prolixus* (from VectorBase)，*Halyomorpha halys* (from NCBI)，*Oncopeltus fasciatus* (from USDA)，*Cimex lectularius* (from NCBI)，and *Drosophila melanogaster* (from NCBI), were aligned to the *T. rubrofasciata* genome with TBLASTN. Full-length transcripts obtained using Iso-Seq were mapped to the genome using Gmap [26]. Finally, gene models predicted from all above methods were combined by MAKER [27], resulting in 12,695 protein-coding genes. The gene number, gene length, CDS length, exon length and intron length distribution were all comparable with the related insects (Figure 4).

To functionally annotate protein-coding genes in the *T. rubrofasciata* genome, we searched all predicted gene sequences to NCBI non-redundant nucleotide (NT) and protein (NR), swiss-prot databases by BLASTN [28] and BLASTX [29]. A threshold of e-value of 1e-5 was used for all BLAST applications. Finally, 12,304 genes were functionally annotated (Table 3).

Phylogenetic analysis of *T. rubrofasciata* with other insects

OrthMCL was used to cluster gene families. First, proteins from *T. rubrofasciata* and the closely related insects, including *Rhodnius prolixus*, *Oncopeltus fasciatus*, *Halyomorpha halys*, *Cimex lectularius*, *Drosophila melanogaster*, *Gerris buenoi*, *Homalodisca vitripennis*, *Acyrthosiphon pisum*，*Culex quinquefasciatus*，*Glossina palpalis*，*Apis mellifera* and *Heliconius melpomene* were all-to-all blasted by BLASTP [29] utility with an e-value threshold of 1e-5. Only proteins from the longest transcript were used for genes with alternative splices. We identified 21,891 gene families for *T. rubrofasciata* and the related species, among them 346 single-copy orthologs families.

Using single-copy orthologs, we could probe the phylogenetic relationships for the *T. rubrofasciata* and other insects. To this end, protein sequences of single-copy genes were aligned using MUSCLE [30]. Guided by the protein multi-sequence alignment, the alignment of the coding DNA sequences (CDS) for those genes were generated and concatenated for the following

analysis. The phylogenetic relationships were constructed using PhyML [31] using the concatenated nucleotide alignment with the JTT+G+F model. The PAML MCMCtree program was used to estimate the species divergent time scales for the insects using approximate likelihood method. We found that *T. rubrofasciata* was most closely related to *R. prolixus*, and the two species diverged from their common ancestor around 51.1-96.2 million years ago (MYA) (Figure 5).

Conclusion

We reconstructed the first chromosome-level assembly of *T. rubrofasciata* using an integrated strategy of PacBio, Illumina and Hi-C technologies. Using the long reads from PacBio Sequel platform and short reads from the Illumina X Ten platform, we successfully constructed contig assembly for *Triatoma*. Leveraging contact information among contigs from Hi-C technology, we further improved the assembly to the chromosome-level quality. We annotated 12,695 protein-coding genes in the *T. rubrofasciata* genome, 12,304 of which were functionally annotated. With 346 single-copy orthologs from *T. rubrofasciata* and other related insects, we construct the phylogenetic relationship of these insects, and found that *T. rubrofasciata* might have diverged from its common ancestor of *R. prolixus* around 51.1-96.2 MYA. Given the increasing interests in insect genome evolution and the biological importance of *T. rubrofasciata* as the vector for Chagas disease, our genomic and transcriptome data provide valuable genetic resource for the following functional genomics investigations for the research community.

Ethics Statement

This study was approved by the Animal Care and Use committee of National Institute of Parasitic Diseases, Chinese Center for Disease Control and Prevention. All participates consent the study under the 'Ethics, consent and permissions' heading. All participants consent to publish the work under the 'Consent to publish' heading.

Funding

This work was supported by the National Key Research and Development Program of China (Grant No. 2016YFC1202000).

Availability of supporting data

The raw data from our genome project was deposited in the NCBI Sequence database with Bioproject IDs PRJNA516044. The Illumina, PacBio and Hi-C sequencing data are available from NCBI via the accession number of SRR8466736, SRR8466737 and SRR8466756, respectively. The Illumina transcriptome sequencing data were deposited to NCBI via the accession number of

SRR8468315 and SRR8468316. The genome, annotation and intermediate files were uploaded to GigaScience FTP server.

Competing interests

The authors declare that they have no competing interests.

Author Contributions

Z.XN, L.Q, Z.Y and H.W conceived the project. L.Q, G.YH, Z.Y, Z.D, L.YY, W.JT and Z.ZB collected the samples and extracted the DNA and RNA. L.Q, G.YH, Z.Y performed the genome assembly and data analysis. C.LS performed the data analysis. L.Q and C.LS wrote the paper. Z.XN revised the manuscript. All authors read, edited and approved the final version of the manuscript.

Acknowledgements

We thank for Frasergen Bioinformatics for providing technique supports for this work.

References

1. Alevi, K.C.C., et al., *Cytogenetic Characterisation of Triatoma rubrofasciata (De Geer) (Hemiptera, Triatominae) Spermatocytes and Its Cytotaxonomic Application.* African Entomology, 2016. **24**(1): p. 4.
2. Hypsa, V., et al., *Phylogeny and biogeography of Triatominae (Hemiptera: Reduviidae): molecular evidence of a New World origin of the Asiatic clade.* Mol Phylogenet Evol, 2002. **23**(3): p. 447-57.
3. Galvão, C., et al., *A checklist of the current valid species of the subfamily Triatominae Jeannel, 1919 (Hemiptera, Reduviidae) and their geographical distribution, with nomenclatural and taxonomic notes.* Zootaxa, 2003. **202**: p. 36.
4. Justi, S.A. and C. GALVÃO, *The Evolutionary Origin of Diversity in Chagas Disease Vectors.* Trends in Parasitology, 2017. **33**(1): p. 11.
5. Carod-Artal, F.J., *Chapter 7 - American trypanosomiasis.* Handbook of Clinical Nerology, 2013. **114**: p. 120.
6. Coura, J.R. and P.A. Vinas, *Chagas disease: a new worldwide challenge.* Nature, 2010. **465**: p. 2.
7. Liu, Q., et al., *First records of Triatoma rubrofasciata (De Geer, 1773) (Hemiptera, Reduviidae) in Foshan, Guangdong Province, Southern China.* Infect Dis Poverty, 2017. **6**(1): p. 129.
8. Neff, K.L., et al., *Mojo Hand, a TALEN design tool for genome editing applications.* BMC Bioinformatics, 2013. **14**: p. 1.
9. Marcais, G. and C. Kingsford, *A fast, lock-free approach for efficient parallel counting of*

occurrences of k-mers. Bioinformatics, 2011. **27**(6): p. 764-70.

10. Mesquita, R.D., et al., *Genome of Rhodnius prolixus, an insect vector of Chagas disease, reveals unique adaptations to hematophagy and parasite infection.* Proc Natl Acad Sci U S A, 2015. **112**(48): p. 14936-41.
11. Chin, C.S., et al., *Phased diploid genome assembly with single-molecule real-time sequencing.* Nat Methods, 2016. **13**(12): p. 1050-1054.
12. Chin, C.S., et al., *Nonhybrid, finished microbial genome assemblies from long-read SMRT sequencing data.* Nat Methods, 2013. **10**(6): p. 563-9.
13. Walker, B.J., et al., *Pilon: an integrated tool for comprehensive microbial variant detection and genome assembly improvement.* PLoS One, 2014. **9**(11): p. e112963.
14. Gong, G., et al., *Chromosomal-level assembly of yellow catfish genome using third-generation DNA sequencing and Hi-C analysis.* Gigascience, 2018. **7**(11).
15. Xu, S., et al., *A draft genome assembly of the Chinese sillago (Sillago sinica), the first reference genome for Sillaginidae fishes.* Gigascience, 2018. **7**(9).
16. Langmead, B., et al., *Ultrafast and memory-efficient alignment of short DNA sequences to the human genome.* Genome Biol, 2009. **10**(3): p. R25.
17. Near, T.J., et al., *Phylogeny and tempo of diversification in the superradiation of spiny-rayed fishes.* Proceedings of the National Academy of Sciences of the United States of America, 2013. **110**(31): p. 12738.
18. English, A.C., et al., *Mind the gap: upgrading genomes with Pacific Biosciences RS long-read sequencing technology.* PLoS One, 2012. **7**(11): p. e47768.
19. Simao, F.A., et al., *BUSCO: assessing genome assembly and annotation completeness with single-copy orthologs.* Bioinformatics, 2015. **31**(19): p. 3210-2.
20. Li, H. and R. Durbin, *Fast and accurate short read alignment with Burrows-Wheeler transform.* Bioinformatics, 2009. **25**(14): p. 1754-60.
21. McKenna, A., et al., *The Genome Analysis Toolkit: a MapReduce framework for analyzing next-generation DNA sequencing data.* Genome Res, 2010. **20**(9): p. 1297-303.
22. Benson, G., *Tandem repeats finder: a program to analyze DNA sequences.* Nucleic Acids Res, 1999. **27**(2): p. 573-80.
23. Bao, W., K.K. Kojima, and O. Kohany, *Repbase Update, a database of repetitive elements in eukaryotic genomes.* Mob DNA, 2015. **6**: p. 11.
24. Chen, N., *Using RepeatMasker to identify repetitive elements in genomic sequences*, in *Current Protocols in Bioinformatics* 2004. p. 14.
25. Stanke, M., et al., *AUGUSTUS: ab initio prediction of alternative transcripts.* Nucleic Acids Res, 2006. **34**(Web Server issue): p. W435-9.
26. Wu, T.D., et al., *GMAP and GSNAP for Genomic Sequence Alignment: Enhancements to Speed, Accuracy, and Functionality.* Methods Mol Biol, 2016. **1418**: p. 283-334.
27. Campbell, M.S., et al., *Genome Annotation and Curation Using MAKER and MAKER-P.* Curr Protoc Bioinformatics, 2014. **48**: p. 4 11 1-39.
28. Gertz, E.M., et al., *Composition-based statistics and translated nucleotide searches: improving the TBLASTN module of BLAST.* BMC Biol, 2006. **4**: p. 41.
29. Camacho, C., et al., *BLAST+: architecture and applications.* BMC Bioinformatics, 2009. **10**: p. 421.
30. Edgar, R.C., *MUSCLE: multiple sequence alignment with high accuracy and high throughput.*

Nucleic Acids Res, 2004. **32**(5): p. 1792-7.
31. Guindon, S. and O. Gascuel, *A simple, fast, and accurate algorithm to estimate large phylogenies by maximum likelihood.* Syst Biol, 2003. **52**(5): p. 696-704.

Tables and Figures

Tables

Table 1: Sequencing data generated for *Triatoma rubrofasciata* genome assembly and annotation

Library type	Platform	Library size (bp)	Data size (Gb)	Application
Short reads	HiSeq X Ten	350	40.96	Genome survey and genomic base correction
Long reads	PacBio SEQUEL	20,000	69.38	Genome assembly
Hi-C	HiSeq X Ten	300-500	99.28	Chromosome construction

Table 2: Statistics for genome assembly of *Triatoma rubrofasciata*

Sample ID	Length		Number	
	Contig** (bp)	Scaffold (bp)	Contig**	Scaffold
Total	680,314,598	680,726,098	2,115	1303
Max	10,270,547	97,329,580	-	-
N50	2,722,109	50,700,875	76	6
N60	2,121,675	50,415,845	104	7
N70	1,587,961	46,556,423	140	8
N80	1,040,245	37,928,883	193	10
N90	343,185	20,341,594	299	12

Table 3: Statistics for genome annotation of *Triatoma rubrofasciata*

Database	Number	Percent
InterPro	9,832	77.45
GO	7,342	57.83
KEGG ALL	11,113	87.54
KEGG KO	6,354	50.05
Swissprot	9,715	76.53
TrEMBL	12,241	96.42
NR	11,697	92.14

Total	12,695

Figures

Figure 1. Dorsal (left) and ventral (right) views of a female *T. rubrofasciata*.

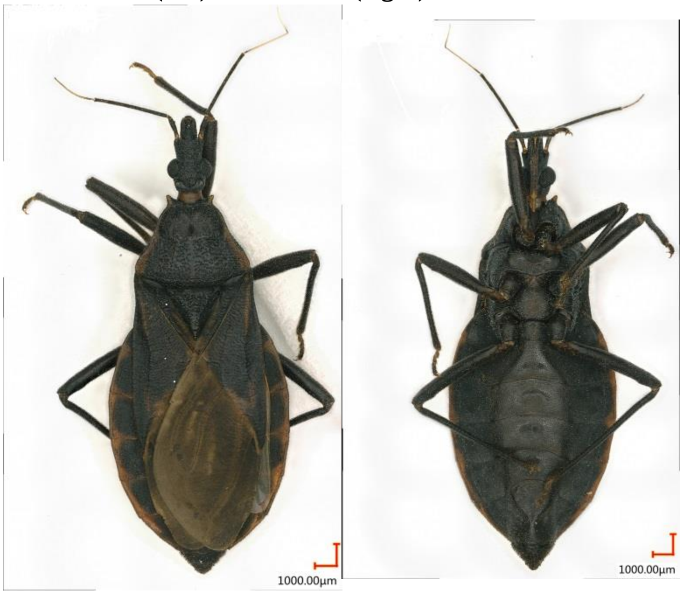

Figure 2. DNA interaction heatmap generated in HiC analysis (resolution: 500 Kb)

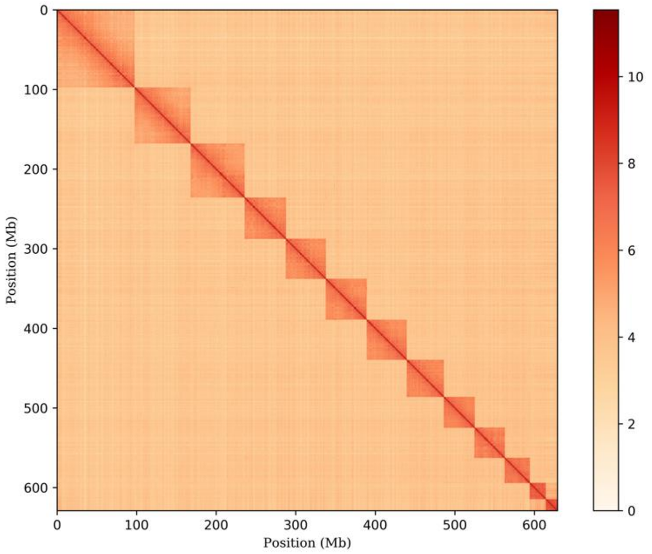

Figure 3: Genome assembly comparison of *T. rubrofasciata* with other sequenced insect genomes. The x- and y-axis represent the contig and scaffold N50s, respectively. The genomes both contig and scaffold N50s less than 2M are highlighted in black.

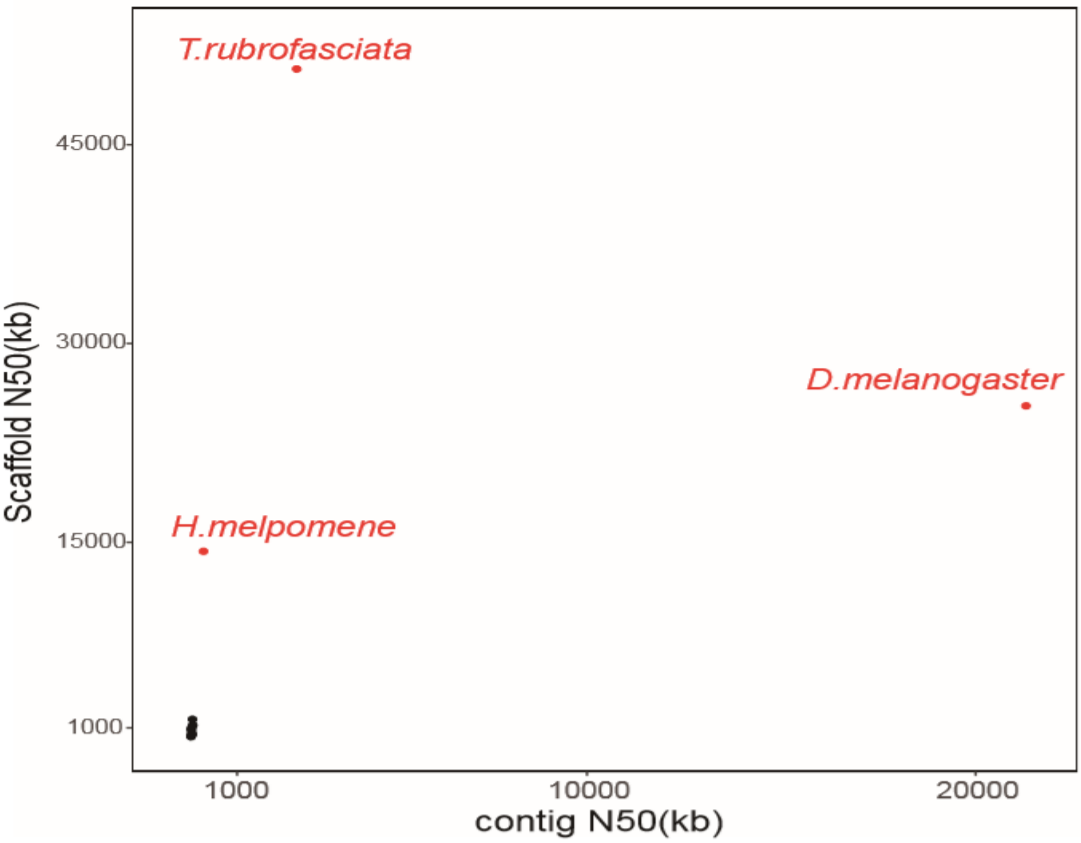

Figure 4: Length distribution comparison on total gene, CDS, exon, and intron of annotated gene models of *T. rubrofasciata* with other closely related insect species. Length distribution of total gene (A), CDS (B), exon (C), and intron (D) were compared to those of *R. prolixus*, *H. halys*, *O. fasciatus*, *C. lectularius* and *D. melanogaster*.

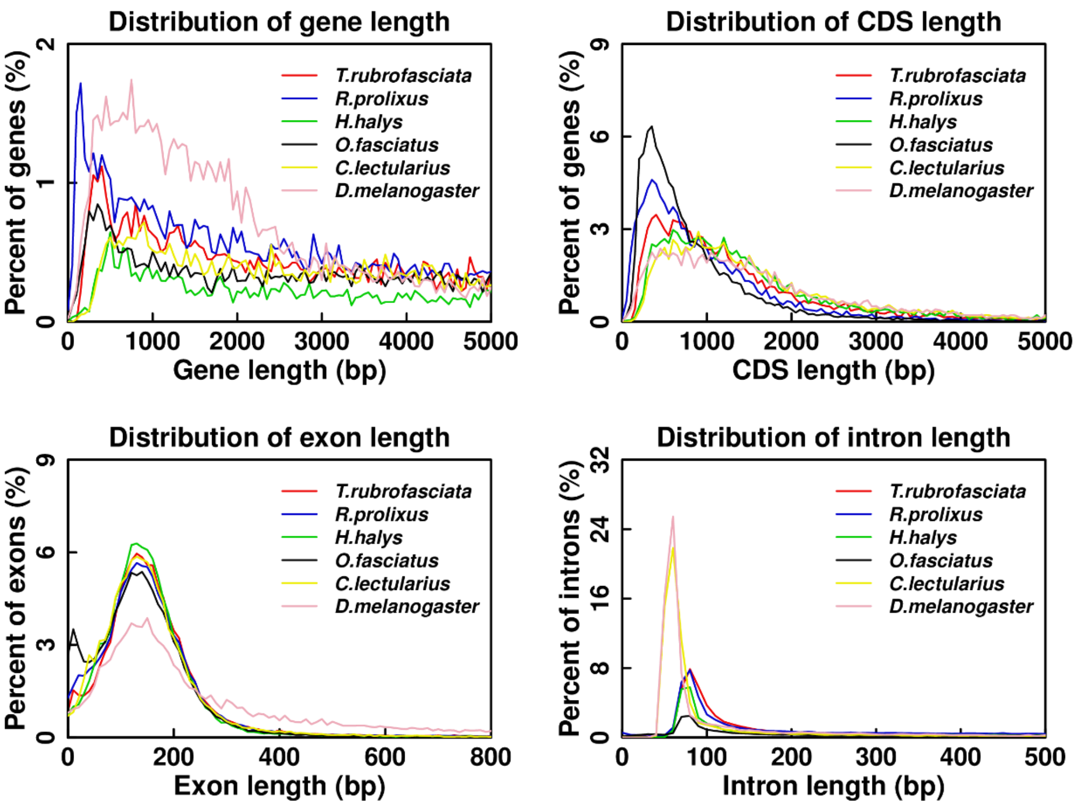

Figure 5: Phylogenetic analysis of *T. rubrofasciata* with other insect species. The estimated species divergence time (million years ago) and the 95% confidential intervals are labeled at each branch site. The divergence used for time recalibration is illuminated as red dots in the tree.

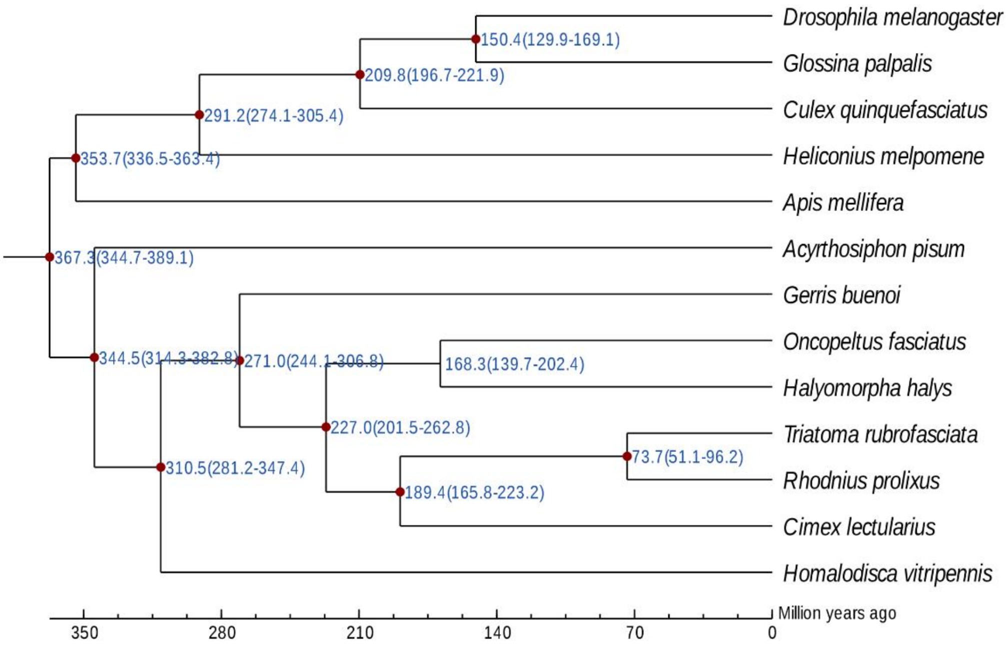

Supplement: giz089_GIGA-D-19-00028_Original_Submission [file giz089_giga-d-19-00028_original_submission.pdf]
