# Supplementary material for: A chromosomal-level genome assembly for the insect vector for Chagas disease, Triatoma rubrofasciata
Source: Gigascience. 2019 Aug 19;8(8):giz089. doi: 10.1093/gigascience/giz089 (PMC6699579; doi:10.1093/gigascience/giz089)
Supplement: giz089_GIGA-D-19-00028_Revision_1 [file giz089_giga-d-19-00028_revision_1.pdf]

GigaScience

A Chromosomal-Level Genome Assembly for the insect vector for Chagas disease, Triatoma rubrofasciata

--Manuscript Draft--

**Manuscript Number:**	GIGA-D-19-00028R1
**Full Title:**	A Chromosomal-Level Genome Assembly for the insect vector for Chagas disease, Triatoma rubrofasciata
**Article Type:**	Data Note
**Funding Information:**	Foundation for the Development of Science and Technology Museums in China (Grant No. 2016YFC1202000) — Prof. Xiao-Nong Zhou
**Abstract:**	Background:<br>Triatoma rubrofasciata is a widespread pathogen vector for Chagas disease, an illness that affects approximately seven million people worldwide. Despite of its importance to human health, its evolutionary origin has not been conclusively determined. A reference genome for T. rubrofasciata is not yet available.<br>Finding:<br>We have sequenced the genome of a female T. rubrofasciata individual using a single molecular DNA sequencing technology (i.e., PacBio Sequel platform) and have successfully reconstructed a whole-genome (680 Mb) assembly that covers 90% of the nuclear genome (757 Mb). Through Hi-C analysis, we have reconstructed full-length chromosomes of this female individual that has 13 unique chromosomes (2n = 24 = 22 + X1 + X2) with a contig N50 of 2.72Mb and a scaffold N50 of 50.7 Mb. This genome has achieved a high base-level accuracy of 99.99%. This platinum-grade genome assembly has 12,691 annotated protein-coding genes. More than 95.1% BUSCO genes were single-copy completed, indicating a high level of completeness of the genome.<br>Conclusion:<br>The platinum-grade genome assembly and its annotation provide valuable information for future in-depth comparative genomics studies including sexual determination analysis in T. rubrofasciata and the pathogenesis of Chagas disease.<br><br>Key Words: Triatoma rubrofasciata, PacBio Sequel platform, Hi-C, chromosomal-level assembly, comparative genomics, RNA-Seq, Iso-Seq
**Corresponding Author:**	Xiao-Nong Zhou<br>National Institute of Parasitic Diseases<br>Shanghai, CHINA
**Corresponding Author Secondary Information:**	
**Corresponding Author's Institution:**	National Institute of Parasitic Diseases
**Corresponding Author's Secondary Institution:**	
**First Author:**	Qin Liu, Ph.D.
**First Author Secondary Information:**	
**Order of Authors:**	Qin Liu, Ph.D.
	Yunhai Guo
	Yi Zhang
	Wei Hu
	Yuanyuan Li
	Dan Zhu

Powered by Editorial Manager® and ProduXion Manager® from Aries Systems Corporation

	Zhengbin Zhou
	Jiatong Wu
	Lansheng Chen
	Nansheng Chen
	Xiao-Nong Zhou
**Order of Authors Secondary Information:**	
**Response to Reviewers:**	Point-to-point responses to Editors of GigaScience<br><br>Dear Editors:<br><br>Thank you very much for sending our manuscript out for review, and we would like to take advantage of this opportunity to thank the reviewers for their constructive comments.<br><br>We have read all comments by the reviewers, and have made corrections to address the issues raised by the reviewers. Most of the suggestions had either been accepted or amended accordingly in this new version. We have also prepared point-to-point responses. Our responses to each comment were written as follows, following each comment in BLUE.<br><br>We hope that our revised manuscript has effectively addressed all comments the reviewers have raised. We appreciated if you find the revised version can be published in GigaScience.<br><br>Sincerely yours,<br><br>Qin Liu (First Author)<br>Xiao-Nong Zhou (Corresponding author)<br><br>Replies to comments:<br>1.Further clarifications on how you obtained the final assembly results are required, as well as the addition of a "genome polishing" sections to further clarify the Hi-C assembly, genome evaluation with BUSCO and the curation procedure used.<br>Re: We have revised the sections including “Genome assembly using PacBio long reads”, “In situ Hi-C library construction and chromosome assembly using Hi-C data”, and “Genome quality evaluation”.<br><br>2.Please ensure that all raw data is accessible - Reviewer #2 was unable to access the raw data for validation.<br>Re: All the raw data have been released at NCBI with the following website address:https://trace.ncbi.nlm.nih.gov/Traces/study/?acc=PRJNA516044&go=go<br><br>Reviewer reports:<br>Reviewer #1:<br>The authors present a very high quality assembly of the genome for Triatoma rubrofasciata that was generated using Illumina X Ten, PacBio and Hi-C libraries. The final genome size reported was 680 Mbp with scaffold and contig N50 values that is superior to most other insect genomes reported to date. The completeness of the genome was estimated at 98% with very few duplicated regions. The genome consists of 12,695 protein coding genes of which 12,304 could be annotated. The gene models compare very well with other insect genomes with regard to gene, CDS, exon and intron length distribution. The data provided with the manuscript adequately cover all features of the study and is accessible in various databases. Some issues follow

Powered by Editorial Manager® and ProduXion Manager® from Aries Systems Corporation

below.
1.Page 1, line 57: … BUSCO genes …
Re: Thank you. We have corrected this data in Page 1 Line 29 and Page 2 Line 1.
2.Page 3, lines 57-60: A plot of the read length distribution/ratio would be useful beyond reporting mean length.
Re: Thanks. We have added a new figure (Figure 2) to illustrate read length distribution.

3.Page 3, line 60: … mean length of reads was 8.43 Kb.
Re: Thanks. We have revised the sentence to "The mean length of these subreads was 8.43 Kb" in Page 4 Line 1.

4.Page 4, line 6: Is reference 8 the appropriate reference for the HTQC package, i.e. Yang et al., 2013?
Re: We thank the reviewer for noting this. We have corrected the reference in the manuscript.

5.Page 4, line 19-22: Independent estimation of the genome size using Kmer analysis is a nice confirmation that the PacBio assembly is correct. Can the Kmer graph be included?
Re: Thanks. We have added a new figure (Figure 3) to show Kmer analysis results.

6.Page 5, line 13: The references for LACHESIS [17] seem to be inappropriate. Reference Burton et al. 2013?
Re: We thank the reviewer for noting this. We have corrected the reference in the manuscript.
7.Page 5, line 35: It is not clear from Figure 3 how many insect genomes are compared since the majority is clustered too close to distinguish between them. Can the genomes be listed in the Figure legend?
Re: Thanks. We have added the names of the insect species in the legend of Figure 5. We have also added an additional attachment in the manuscript named N50.

namescaffold N50(bp)contig N50(bp)address
A.mellifera120894949814ftp://ftp.hgsc.bcm.edu/Amellifera/Genes/Amel_4.5_OGSv3.2/
A.pisum51854628192http://bipaa.genouest.org/data/public/a_pisum/
C.lectularius163764442565https://www.ncbi.nlm.nih.gov/assembly/GCF_000648675.2
C.quinquefasciatus48675628546ftp://ftp.ncbi.nlm.nih.gov/genomes/all/GCF/000/209/185/GCF_000209185.1_CulPip1.0
D.melanogaster2528693621485538https://www.ncbi.nlm.nih.gov/assembly/GCF_000001215.4
G.buenoi3441183812ftp://ftp.hgsc.bcm.edu/I5K-pilot/Water_strider/maker_annotation/version_0.5.3/
G.palpalis57503721728https://www.vectorbase.org/download/
H.halys80242317705https://www.ncbi.nlm.nih.gov/assembly/GCF_000696795.2
H.melpomene14308859324837http://download.lepbase.org/v4/
H.vitripennis9140094858ftp://ftp.hgsc.bcm.edu/I5K-pilot/Glassy-winged_sharpshooter/maker_annotation/version_0.5.3/
O.fasciatus3399604047https://usdasearch.usda.gov/search?utf8=%E2%9C%93&affiliate=usda&query=Oncopeltus+fasciatus&commit=Search
R.prolixus108877234095https://www.vectorbase.org/taxonomy/rhodnius-prolixus

8.Page 5, line 41: Not sure why sentence refers to difficulty of mollusk genome assembly? It seems as if parts of the manuscript were copied from another manuscript without updating key words or references?
Re: We thank the reviewer for spotting this. It was the unpublished work we have done in mollusk genome. We have rewritten this paragraph.

9.Page 7, lines 1-8: The phylogenetic analysis needs more information. How was the nodes calibrated for the molecular clock analysis? Include specific fossil data used for

Powered by Editorial Manager® and ProduXion Manager® from Aries Systems Corporation

	calibration. It should be noted that the date estimates for the divergence of Rhodnius/Triatoma (51-96 MYA) is much older than other molecular clock estimates (Hwang and Weirauch, 2012). In fact the upper estimate is almost as old the estimate for the higher Reduviidae as a group. The authors should at least address these differences.<br>Re: Thanks. We have revised the text by adding more details regarding the analysis. We added these text in Page 7 Lines 6-10: "We first obtained divergent times for all pair using the phylogenetic tree using r8s (Sanderson et al., 2003), which were used as input, together with pair-wise fossil calibration time from TimeTree (Kumer et al., 2017), to estimate species divergence time for all pairs of species in the phylogenetic tree using MCMCtree program (from PAML) (Yang et al., 1997)."<br><br>Reviewer #2: The authors sequenced a female blood-sucking insect Triatoma rubrofasciata, which is a pathogen vector of Chagas disease.<br><br>With PacBio sequencing, they reconstructed an assembly covering 99% of the 667 Mb genome, and used Hi-C analysis to reconstruct 13 haploid full-length chromosomes with a contig N50 near 3 Mb and a scaffold N50 over 50 Mb. The authors claimed a base-accuracy of 99.99%. More than 12k protein coding genes has been annotated with 97% BUSCO score that suggests a high genome completeness.<br>Re: Thank you very much.<br><br>The methods employed and the description in the study are mostly appropriate and standard. The integration of long-read PacBio sequencing with Hi-C analysis for chromosome reconstruction has become one of the standard pipeline for de novo genome assembly nowadays. The choice of a diploid female individual is suitable for a species without prior quality reference. Key global statistics numbers, including total length, max length and N50 of contigs and scaffolds listed in Table 2 are validated. However, there are some obvious confusion in obtaining the final assembly results. The scaffold N50 is mentioned in text several times as 51.38 Mb, while it is 50,700,875 bp in Table 2, as well as checked with the data uploaded. Similarly, contig N50 is 2.96 Mb in text, and 2,722,109 in Table 2 and data. It is unclear how the assemblies resolve from Falcon-assembly with 2,115 contigs and Hi-C assembly with 626 contigs, into the final assembly with 1,303 scaffolds. The authors should add<br>a section of "genome polishing" between Hi-C assembly and genome evaluation with BUSCO to describe the reconciliation process, or at least mention of a curation procedure. For BUSCO genome evaluation, the authors should also specify which reference gene set was used）.<br>Re: Thank you. We have revised the manuscript to clarify these numbers, and have added necessary references.<br><br>1.Key global statistics numbers, including total length, max length and N50 of contigs and scaffolds listed in Table 2.<br>Re：Thanks. We have checked and corrected the numbers in Table 2.<br><br>2.It is unclear how the assemblies resolve from Falcon-assembly with 2,115 contigs and Hi-C assembly with 626 contigs, into the final assembly with 1,303 scaffolds<br>Re：Thanks. We have checked the data and rewritten those in Page 5, Lines 4-15. The assembled genome consisted of 1,030 scaffolds, which included 13 chromosomes and 1,290 unanchored scaffolds.<br><br>3.For BUSCO genome evaluation, the authors should also specify which reference gene set was used.<br>Re：We have added the reference gene set information in the manuscript.<br><br>4.The authors should add a section of "genome polishing" between Hi-C assembly and genome evaluation with BUSCO to describe the reconciliation process, or at least mention of a curation procedure.<br>Re：Thanks. We have added description about polishing in the "Genome assembly using PacBio long reads" section. We have added the reference gene set information for BUSCO analysis.<br><br>5.In addition, the unavailability of raw data and specific parameters, including the scores and thresholds for alignment and phylogenetic tree construction prevents

	validation. In the last section of methods on constructing the phylogenetic tree, the authors should state the source of sequences of other insects, as well as using an outgroup. Therefore, the validity of the authors' claim of species divergence time cannot be assessed.<br>Re: Thanks. We have submitted the data to NCBI and accession numbers are included in the revised manuscript. The source of sequences for other insects was included in a new file.
Additional Information:	
Question	Response
Are you submitting this manuscript to a special series or article collection?	No
**Experimental design and statistics**<br><br>Full details of the experimental design and statistical methods used should be given in the Methods section, as detailed in our Minimum Standards Reporting Checklist. Information essential to interpreting the data presented should be made available in the figure legends.<br><br>Have you included all the information requested in your manuscript?	Yes
**Resources**<br><br>A description of all resources used, including antibodies, cell lines, animals and software tools, with enough information to allow them to be uniquely identified, should be included in the Methods section. Authors are strongly encouraged to cite Research Resource Identifiers (RRIDs) for antibodies, model organisms and tools, where possible.<br><br>Have you included the information requested as detailed in our Minimum Standards Reporting Checklist?	Yes
**Availability of data and materials**<br><br>All datasets and code on which the conclusions of the paper rely must be either included in your submission or deposited in publicly available repositories	Yes

Powered by Editorial Manager® and ProduXion Manager® from Aries Systems Corporation

(where available and ethically appropriate), referencing such data using a unique identifier in the references and in the “Availability of Data and Materials” section of your manuscript.<br><br>Have you have met the above requirement as detailed in our Minimum Standards Reporting Checklist?	

Revised version of the manuscript
Click here to view linked References
Click here to access/download;Manuscript;Triatoma rubrofasciata genome-clean version.docx

A Chromosomal-Level Genome Assembly for the insect vector for Chagas disease, *Triatoma rubrofasciata*

Qin Liu[1#], Yunhai Guo[1#], Yi Zhang[1#], Wei Hu[1,2] ,Yuanyuan Li[1], Dan Zhu[1], Zhengbin Zhou[1], Jiatong Wu[1], Nansheng Chen[3,4,5*], Xiao-Nong Zhou[1*]

[1]National Institute of Parasitic Diseases, Chinese Center for Disease Control and Prevention; Key Laboratory of Parasite and Vector Biology, Ministry of Health; WHO Collaborating Center for Tropical Diseases; Chinese Center for Tropical Diseases Research, Shanghai 200025, P. R. China

[2]Department of Microbiology and Microbial Engineering, School of Life Sciences，Fudan, Shanghai 200025, P. R. China

[3]CAS Key laboratory of Marine Ecology and Environmental Sciences, Institute of Oceanology, Chinese Academy of Sciences; Qingdao, Shandong 266071, China；

[4]Laboratory for Marine Ecology and Environmental Science, Qingdao National Laboratory for Marine Science and Technology, Qingdao, Shandong 266237, China；

[5]Department of Molecular Biology and Biochemistry, Simon Fraser University, Burnaby, Canada

Abstract

Background:

Triatoma rubrofasciata is a widespread pathogen vector for Chagas disease, an illness that affects approximately seven million people worldwide. Despite of its importance to human health, its evolutionary origin has not been conclusively determined. A reference genome for *T. rubrofasciata* is not yet available.

Finding:

We have sequenced the genome of a female *T. rubrofasciata* individual using a single molecular DNA sequencing technology (i.e., PacBio Sequel platform) and have successfully reconstructed a whole-genome (680 Mb) assembly that covers 90% of the nuclear genome (757 Mb). Through Hi-C analysis, we have reconstructed full-length chromosomes of this female individual that has 13 unique chromosomes (2n = 24 = 22 + X1 + X2) with a contig N50 of 2.72Mb and a scaffold N50 of 50.7 Mb. This genome has achieved a high base-level accuracy of 99.99%. This platinum-grade genome assembly has 12,691 annotated protein-coding genes. More than 95.1%

BUSCO genes were single-copy completed, indicating a high level of completeness of the genome.

Conclusion:

The platinum-grade genome assembly and its annotation provide valuable information for future in-depth comparative genomics studies including sexual determination analysis in *T. rubrofasciata* and the pathogenesis of Chagas disease.

Key Words: *Triatoma rubrofasciata*, PacBio Sequel platform, Hi-C, chromosomal-level assembly, comparative genomics, RNA-Seq, Iso-Seq

Data description

Introduction

The insect *T. rubrofasciata* (De Geer) (Hemiptera, Triatominae) is the first Triatominae species formally described, initially with the name *Cimex rubrofasciatus* De Geer, 1773 [1]. This insect presents anthropogenic habits with its dispersion favored by the interaction between residential settlement and human activities [2]. It is considered of global epidemiological importance since it has a pantropical widespread distribution which is found in approximately 45 countries from the Old World to the New World [3]. It is one of the 151 species of Triatominae that has 18 genera currently described worldwide that can transmit American trypanosomiasis known as Chagas disease [4]. This condition has great impact on public health, with 7-8 million people estimated to be infected worldwide, mostly in Latin America. It has become a global health issue in this century with the spread to the non-endemic countries due to growing population movements [5].

Due to growing population movements, important epidemiological changes have occurred in recent decades, and the disease has now spread to many non-endemic countries [6] The widespread of *T. rubrofasciata* emerges as a potential risk of outbreaks in these regions, which demands urgent studies through comprehensive sampling and comparative studies. The lack of a high-quality reference genome represents a major hurdle for such efforts. Here, we present a platinum-grade reference genome for *T. rubrofasciata*, which will be valuable for developing vector control programs.

Sample description and DNA sequencing

An adult female insect *T. rubrofasciata* (Figure 1) was used for reference genome construction in this study. This insect was the second generation offspring of a population that was established from the eggs of single female adult collected in Shunde County, Foshan City, Guangdong Province (22°42′44.63″N, 113°08′45.34″E), China, in 2016 [7]. DNA was extracted from this individual using the traditional phenol/chloroform extraction method and was quality checked using agarose gel electrophoresis. A single band was observed, indicating high integrity of DNA molecules for library construction for the Illumina X Ten (Illumina Inc., San Diego, CA, USA) and the PacBio Sequel (Pacific Biosciences of California, Menlo Park, CA, USA) sequencing platforms.

Using the DNA preparation, a library with the insertion length of 350 bp was constructed for Illumina sequencing platform according to the manufacturer's protocol. 46.75 Gb short reads were obtained from the Illumina X Ten DNA sequencing platform (Table 1). 39.32 Gb filtered reads were used for the following genome survey analysis, and for final-stage base-level genome sequence polishing. Meanwhile, 20 Kb-libraries were constructed for PacBio Sequel sequencing. Using fourteen SMRT cells, 8.23 million reads were generated, with the total length of 69.38 Gb

(Table 1). The mean length of these subreads was 8.43 Kb and the plot of the read length distribution/ratio was showing in Figure 2.

Genome features estimation through *K*mer analysis

With sequencing data from the Illumina HiSeq X Ten DNA sequencing platform, several genome features were evaluated for the genome of *T. rubrofasciata*. To ensure the quality of the analysis, ambiguous bases and low-quality reads were first trimmed and filtered using the HTQC package [8]. First, the quality of bases at two read ends was checked. Bases in sliding 5 bp windows were deleted if the average quality of the window was below 20. Second, reads were filtered if the average quality were smaller than 20 or the read length was shorter than 75 bp. Third, the mate reads were also removed if the corresponding reads were filtered.

The processed reads were used for genome assessment. We calculated the number of each 17-mer from the sequencing data using the jellyfish software (v2.1.3) [9], and the distribution was analyzed with GCE software. We estimated the genome size of 757 Mb with the heterozygosity of 1.01% and repeat content of 55.49% in the genome. Kmer analysis was using to estimate the genome size which showed the PacBio assembly was of good quality (Figure 3). The genome size of *T. rubrofasciata* is similar to that of *Rhodnius prolixus*, another insect vector of Chagas disease, which has a predicted 733 Mb genome size [10].

Genome assembly using PacBio long reads

FALCON [11] was employed using the length_cut_off and length_cutoff_pr parameters of 3 Kb and 3 Kb, respectively. We first obtained 677.72 Mb genome with a contig N50 of 2.71 Mb. The genome sequences were subsequently polished using PacBio long reads using arrow [12] and Illumina short reads by pilon [13] to correct base errors.

In situ Hi-C library construction and chromosome assembly using Hi-C data

A separate female individual *T. rubrofasciata* was used for library construction for Hi-C analysis as described previously [14, 15]. The library was sequenced with 150 bp paired-end mode on the Illumina HiSeq X Ten platform (San Diego, CA, United States).

From the Illumina HiSeq X Ten platform, 103.61 Gb reads were obtained for the Hi-C library and 99.28 Gb filtered reads were used for the following Hi-C analysis. The reads were mapped to the above *T. rubrofasciata* genome with Bowtie [16], with both ends of paired reads being mapped to the genome separately. To increase the interactive Hi-C reads ratio, an iterative mapping strategy was performed as previous studies, and only read pairs that both ends uniquely mapped were used for the following analysis. From the alignment of the paired ends, self-ligation, non-ligation and other sorts of invalid reads, including StartNearRsite, PCR amplification, random

break, LargeSmallFragments and ExtremeFragments, were filtered out by Hi-C lib and the method was described in a previous study [14]. Through the recognition of restriction sites in sequences, contact counts among contigs were calculated and normalized.

By clustering the contigs using the contig contact frequency matrix, we were able to correct some minor errors in the FALCON assembly results. Contigs with errors were corrected by breaking into shorter contigs, we obtained a chromosome-level genome assembly of 680.73 Mb with 2,126 contigs, and a contig N50 of 2.72 Mb. The longest contig was 10.27 Mb in size (Table 2). Among these 2,126 contigs, 626 contigs were mounted to 13 chromosomes with Lachesis [17] using the agglomerative hierarchical clustering method. Lachesis was further applied to order and orient the clustered contigs according to the contact matrix. Contigs anchored to chromosomes accounted for 92.51% of the total genome bases (Figure 4). The number of chromosomes matched nicely to previously published karyotype of a female *T. rubrofasciata* individual (2n = 24 = 11 * 2 + X1 + X2) [1]. Taken together, we have successfully reconstructed the first chromosomal-level assembly of *T. rubrofasciata* of 680.73 Mb, with 2,126 contigs, a contig N50 of 2.72 Mb, a scaffold N50 of 50.70 Mb (Table 2).

Genome quality evaluation

We assessed the quality of genome of *T. rubrofasciata* in three aspects: sequence continuity, genome completeness and base level accuracy.

First of all, we compared the contig/scaffold number and N50 length of contig of *T. rubrofasciata* with insect species with those of sequenced genomes and found that our assembly has much improved quality over other insects (Figure 5). We attributed the advantage to the application of the PacBio long reads for genome assembly. With Hi-C data analysis, we successfully assembled *T. rubrofasciata* genome into chromosome-level with just one individual. As previous studies, genomic heterozygosity of insects was one of the biggest challenges for genome assembly, both in terms of contig and scaffold assembly. Traditional chromosomal genome assembly requires physical maps and genetic maps, which is enormously time- and labor-consuming. Our work illustrated that the genome assembly using PacBio long sequencing data was not only affordable but also effective for overcoming the difficulty of mollusk genome assembly.

Second, the assembled genome was subjected to the BUSCO v.3.0.2 (Benchmarking Universal Single-Copy Orthologs, RRID:SCR_015008) [18] to assess the completeness of the genome assembly. We used “insect_obd9” gene set. 98.2% of the BUSCO genes were identified in *T. rubrofasciata* genome. More than 95.1% BUSCO gene were single-copy completed in our genome, illuminating a high level of completeness of the genome.

Third, NGS short reads were aligned to the genome using BWA [19]. About 98.1% of reads were aligned to the genome, of which 98.0% were reads paired aligned. The insertion length

distribution of read pairs exhibited a single peak around 300 bp, which was consistent with the design for the Illumina sequencing library construction. Note that the NGS data, which was used for error correction, was not used in contig assembly. Therefore, the insertion length distribution of NGS data illustrated the high quality of our assembly at the contig level. From the NGS reads alignment, we detected 8,478 homologous SNP loci using GATK [20], demonstrating the high base-level accuracy of 99.99%.

Repeat element and gene annotation

Tandem Repeat Finder (TRF) [21] was used for repetitive element identification in *T. rubrofasciata* genome. A *de novo* method applying RepeatModuler (http://www.repeatmasker.org/RepeatModeler.html) was used to detect transposable elements (TEs). The resulting *de novo* data, combined with known repeat library from Repbase [22], were used to identify TEs in the *T. rubrofasciata* genome by RepeatMasker [23].

Protein-coding genes in the *T. rubrofasciata* genome were annotated using the *de novo* program Augustus (RRID:SCR_008417) [24]. Protein sequences of the closely related species including *Rhodnius prolixus* (from VectorBase)，*Halyomorpha halys* (from NCBI)，*Oncopeltus fasciatus* (from USDA)，*Cimex lectularius* (from NCBI)，and *Drosophila melanogaster* (from NCBI), were aligned to the *T. rubrofasciata* genome with tblastn. Full-length transcripts obtained using Iso-Seq were mapped to the genome using Gmap [25]. Finally, gene models predicted from all above methods were combined by MAKER [26], resulting in 12,691 protein-coding genes. The gene number, gene length, CDS length, exon length and intron length distribution were all comparable with the related insects (Figure 6).

To functionally annotate protein-coding genes in the *T. rubrofasciata* genome, we searched all predicted gene sequences to NCBI non-redundant protein (NR), InterPro (InterProScan, RRID:SCR_005829) [27], GO (Gene Ontology), KEGG (RRID:SCR_012773) [28], Swissprot [29], TrEMBL databases [29] by BLASTN [30] and BLASTX [31]. A threshold of e-value of 1e-5 was used for all BLAST applications. Finally, 12,063 genes were functionally annotated (Table 3).

Phylogenetic analysis of *T. rubrofasciata* with other insects

OrthMCL was used to cluster gene families. First, proteins from *T. rubrofasciata* and the closely related insects, including *Rhodnius prolixus*, *Oncopeltus fasciatus*, *Halyomorpha halys*, *Cimex lectularius*, *Drosophila melanogaster*, *Gerris buenoi*, *Homalodisca vitripennis*, *Acyrthosiphon pisum*，*Culex quinquefasciatus*，*Glossina palpalis*，*Apis mellifera* and *Heliconius melpomene* were all-to-all blasted by BLASTP [31] utility with an e-value threshold of 1e-5. Only proteins from the longest transcript were used for genes with alternative splices. We identified 21,850 gene families for *T. rubrofasciata* and the related species, among them 330 single-copy orthologs families.

Using single-copy orthologs, we probed the phylogenetic relationships for the *T.*

rubrofasciata and other insects. To this end, protein sequences of single-copy genes were aligned using MUSCLE [32]. Guided by the protein multi-sequence alignment, the alignment of the coding DNA sequences (CDS) for those genes were generated and concatenated for the following analysis. The phylogenetic relationships were constructed using PhyML [33] using the concatenated nucleotide alignment with the JTT+G+F model. We first obtained divergent times for all pair using the phylogenetic tree using r8s [34], which were used as input, together with molecular clock data from the divergence time from the TimeTree database [35], to estimate species divergence time for all pairs of species in the phylogenetic tree using MCMCtree program (from PAML) [36]. We found that *T. rubrofasciata* was most closely related to *R. prolixus*, and the two species diverged from their common ancestor around 60.00-95.00 million years ago (MYA) (Figure 7).

Conclusion

We reconstructed the first high-quality, chromosome-level assembly of *T. rubrofasciata* using an integrated strategy of PacBio, Illumina and Hi-C technologies. Using the long reads from PacBio Sequel platform and short reads from the Illumina HiSeq X Ten platform, we successfully constructed contig assembly for *Triatoma*. Leveraging contact information among contigs from Hi-C technology, we further improved the assembly to the chromosome-level quality. We annotated 12,691 protein-coding genes in the *T. rubrofasciata* genome, 12,063 of which were functionally annotated. With 330 single-copy orthologs from *T. rubrofasciata* and other related insects, we construct the phylogenetic relationship of these insects, and found that *T. rubrofasciata* might have diverged from its common ancestor of *R. prolixus* around 60.00-95.00 MYA. Given the increasing interests in insect genome evolution and the biological importance of *T. rubrofasciata* as the vector for Chagas disease, our genomic and transcriptome data provide valuable genetic resource for the following functional genomics investigations for the research community.

Ethics Statement

This study was approved by the Animal Care and Use committee of National Institute of Parasitic Diseases, Chinese Center for Disease Control and Prevention. All participates consent the study under the 'Ethics, consent and permissions' heading. All participants consent to publish the work under the 'Consent to publish' heading.

Funding

This work was supported by the National Key Research and Development Program of China (Grant No. 2016YFC1202000)，the National Science and Technology Project (No. 201810101002)

and the CAS Pioneer Hundred Talents Program (to N.S.C.) and Taishan Scholar Project Special Fund (to N.S.C.).

Availability of supporting data

The raw data from our genome project was deposited in the NCBI Sequence database with Bioproject IDs PRJNA516044. The Illumina, PacBio and Hi-C sequencing data are available from NCBI via the accession number of SRR8466736, SRR8466737 and SRR8466756, respectively. The Illumina transcriptome sequencing data were deposited to NCBI via the accession number of SRR8468315 and SRR8468316. The genome, annotation and intermediate files were uploaded to GigaScience FTP server.

Competing interests

The authors declare that they have no competing interests.

Author Contributions

Z.X.N., L.Q., Z.Y. and H.W. conceived the project. L.Q., G.Y.H., Z.Y., Z.D., L.Y.Y., W.J.T. and Z.Z.B. collected the samples and extracted the DNA and RNA. L.Q, G.Y.H., Z.Y. performed the genome assembly and data analysis. C.N.S. performed the data analysis. L.Q and C.N.S. wrote the paper. Z.X.N. revised the manuscript. All authors read, edited and approved the final version of the manuscript.

Acknowledgements

We thank for Frasergen Bioinformatics for providing technique supports for this work.

References

1. Alevi, K.C.C., et al., *Cytogenetic Characterisation of Triatoma rubrofasciata (De Geer) (Hemiptera, Triatominae) Spermatocytes and Its Cytotaxonomic Application.* African Entomology, 2016. **24**(1): p. 4.
2. Hypsa, V., et al., *Phylogeny and biogeography of Triatominae (Hemiptera: Reduviidae): molecular evidence of a New World origin of the Asiatic clade.* Mol Phylogenet Evol, 2002. **23**(3): p. 447-57.
3. Galvão, C., et al., *A checklist of the current valid species of the subfamily Triatominae Jeannel, 1919 (Hemiptera, Reduviidae) and their geographical distribution, with nomenclatural and taxonomic notes.* Zootaxa, 2003. **202**: p. 36.
4. Justi, S.A. and C. GALVÃO, *The Evolutionary Origin of Diversity in Chagas Disease Vectors.* Trends in Parasitology, 2017. **33**(1): p. 11.

5. Carod-Artal, F.J., *Chapter 7 - American trypanosomiasis.* Handbook of Clinical Nerology, 2013. **114**: p. 120.
6. Coura, J.R. and P.A. Vinas, *Chagas disease: a new worldwide challenge.* Nature, 2010. **465**: p. 2.
7. Liu, Q., et al., *First records of Triatoma rubrofasciata (De Geer, 1773) (Hemiptera, Reduviidae) in Foshan, Guangdong Province, Southern China.* Infect Dis Poverty, 2017. **6**(1): p. 129.
8. Neff, K.L., et al., *Mojo Hand, a TALEN design tool for genome editing applications.* BMC Bioinformatics, 2013. **14**: p. 1.
9. Marcais, G. and C. Kingsford, *A fast, lock-free approach for efficient parallel counting of occurrences of k-mers.* Bioinformatics, 2011. **27**(6): p. 764-70.
10. Mesquita, R.D., et al., *Genome of Rhodnius prolixus, an insect vector of Chagas disease, reveals unique adaptations to hematophagy and parasite infection.* Proc Natl Acad Sci U S A, 2015. **112**(48): p. 14936-41.
11. Chin, C.S., et al., *Phased diploid genome assembly with single-molecule real-time sequencing.* Nat Methods, 2016. **13**(12): p. 1050-1054.
12. Chin, C.S., et al., *Nonhybrid, finished microbial genome assemblies from long-read SMRT sequencing data.* Nat Methods, 2013. **10**(6): p. 563-9.
13. Walker, B.J., et al., *Pilon: an integrated tool for comprehensive microbial variant detection and genome assembly improvement.* PLoS One, 2014. **9**(11): p. e112963.
14. Gong, G., et al., *Chromosomal-level assembly of yellow catfish genome using third-generation DNA sequencing and Hi-C analysis.* Gigascience, 2018. **7**(11).
15. Xu, S., et al., *A draft genome assembly of the Chinese sillago (Sillago sinica), the first reference genome for Sillaginidae fishes.* Gigascience, 2018. **7**(9).
16. Langmead, B., et al., *Ultrafast and memory-efficient alignment of short DNA sequences to the human genome.* Genome Biol, 2009. **10**(3): p. R25.
17. Near, T.J., et al., *Phylogeny and tempo of diversification in the superradiation of spiny-rayed fishes.* Proceedings of the National Academy of Sciences of the United States of America, 2013. **110**(31): p. 12738.
18. Simao, F.A., et al., *BUSCO: assessing genome assembly and annotation completeness with single-copy orthologs.* Bioinformatics, 2015. **31**(19): p. 3210-2.
19. Li, H. and R. Durbin, *Fast and accurate short read alignment with Burrows-Wheeler transform.* Bioinformatics, 2009. **25**(14): p. 1754-60.
20. McKenna, A., et al., *The Genome Analysis Toolkit: a MapReduce framework for analyzing next-generation DNA sequencing data.* Genome Res, 2010. **20**(9): p. 1297-303.
21. Benson, G., *Tandem repeats finder: a program to analyze DNA sequences.* Nucleic Acids Res, 1999. **27**(2): p. 573-80.
22. Bao, W., K.K. Kojima, and O. Kohany, *Repbase Update, a database of repetitive elements in eukaryotic genomes.* Mob DNA, 2015. **6**: p. 11.
23. Chen, N., *Using RepeatMasker to identify repetitive elements in genomic sequences*, in *Current Protocols in Bioinformatics* 2004. p. 14.
24. Stanke, M., et al., *AUGUSTUS: ab initio prediction of alternative transcripts.* Nucleic Acids Res, 2006. **34**(Web Server issue): p. W435-9.
25. Wu, T.D., et al., *GMAP and GSNAP for Genomic Sequence Alignment: Enhancements to Speed, Accuracy, and Functionality.* Methods Mol Biol, 2016. **1418**: p. 283-334.

26. Campbell, M.S., et al., *Genome Annotation and Curation Using MAKER and MAKER-P.* Curr Protoc Bioinformatics, 2014. **48**: p. 4 11 1-39.
27. Quevillon, E., et al., *InterProScan: protein domains identifier.* Nucleic Acids Res, 2005. **33**(Web Server issue): p. W116-20.
28. Kanehisa, M. and S. Goto, *KEGG: kyoto encyclopedia of genes and genomes.* Nucleic Acids Res, 2000. **28**(1): p. 27-30.
29. Boeckmann, B., et al., *The SWISS-PROT protein knowledgebase and its supplement TrEMBL in 2003.* Nucleic Acids Res, 2003. **31**(1): p. 365-70.
30. Gertz, E.M., et al., *Composition-based statistics and translated nucleotide searches: improving the TBLASTN module of BLAST.* BMC Biol, 2006. **4**: p. 41.
31. Camacho, C., et al., *BLAST+: architecture and applications.* BMC Bioinformatics, 2009. **10**: p. 421.
32. Edgar, R.C., *MUSCLE: multiple sequence alignment with high accuracy and high throughput.* Nucleic Acids Res, 2004. **32**(5): p. 1792-7.
33. Guindon, S. and O. Gascuel, *A simple, fast, and accurate algorithm to estimate large phylogenies by maximum likelihood.* Syst Biol, 2003. **52**(5): p. 696-704.
34. Sanderson, M.J., *r8s: inferring absolute rates of molecular evolution and divergence times in the absence of a molecular clock.* Bioinformatics, 2003. **19**(2): p. 301-2.
35. Kumar, S., et al., *TimeTree: A Resource for Timelines, Timetrees, and Divergence Times.* Mol Biol Evol, 2017. **34**(7): p. 1812-1819.
36. Yang, Z., *PAML: a program package for phylogenetic analysis by maximum likelihood.* Computer Applications in the Biosciences, 1997. **13**(5): p. 2.

Tables and Figures

Tables

Table 1: Sequencing data generated for *Triatoma rubrofasciata* genome assembly and annotation

Library type	Platform	Library size (bp)	Data size (Gb)	Application
Short reads	HiSeq X Ten	350	46.75	Genome survey and genomic base correction
Long reads	PacBio Sequel	20,000	69.38	Genome assembly
Hi-C	HiSeq X Ten	300-500	103.61	Chromosome construction

Table 2: Statistics for genome assembly of *Triatoma rubrofasciata*

Sample ID	Length		Number	
	Contig** (bp)	Scaffold (bp)	Contig**	Scaffold
Total	680,314,598	680,726,098	2,126	1,303
Max	10,270,547	97,329,580	-	-
N50	2,722,109	50,700,875	76	6
N60	2,121,675	50,415,845	104	7
N70	1,587,961	46,556,423	140	8
N80	1,038,484	37,928,883	193	10
N90	338,786	20,341,594	301	12

Table 3: Statistics for genome annotation of *Triatoma rubrofasciata*

Database	Number	Percent
NR	11,451	90.23
InterPro	9,625	75.84
GO	7,180	56.58
KEGG ALL	10,867	85.63
KEGG KO	6,112	48.16
Swissprot	9,448	74.45
TrEMBL	11,989	94.47
Total	12,063	95.05

Figures

Figure 1. Dorsal (left) and ventral (right) views of a female *T. rubrofasciata*.

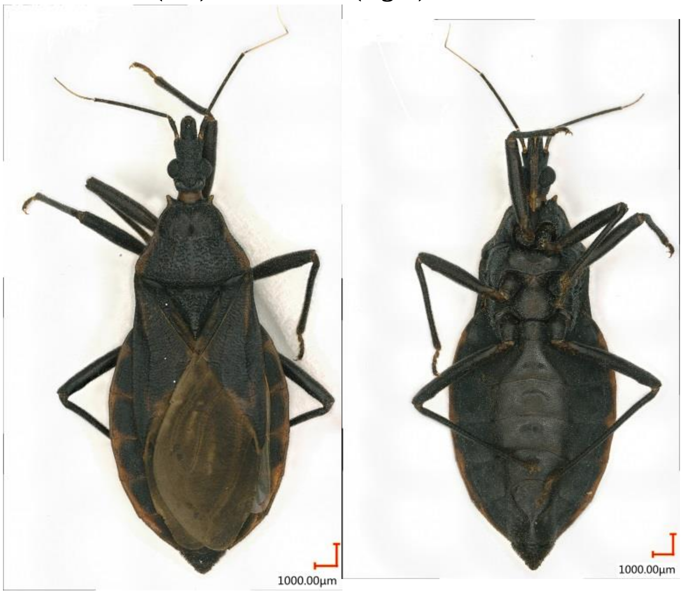

Figure 2. The plot of the read length distribution/ratio of the subreads.

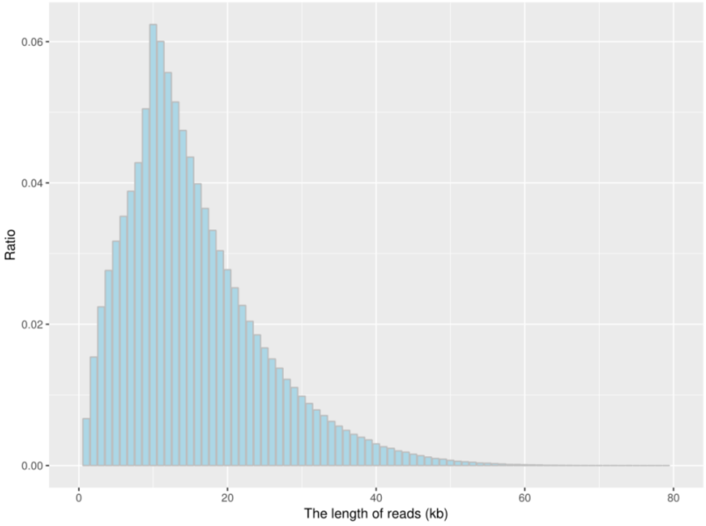

Figure 3. Kmer analysis of the genome size of *T. rubrofasciata*.

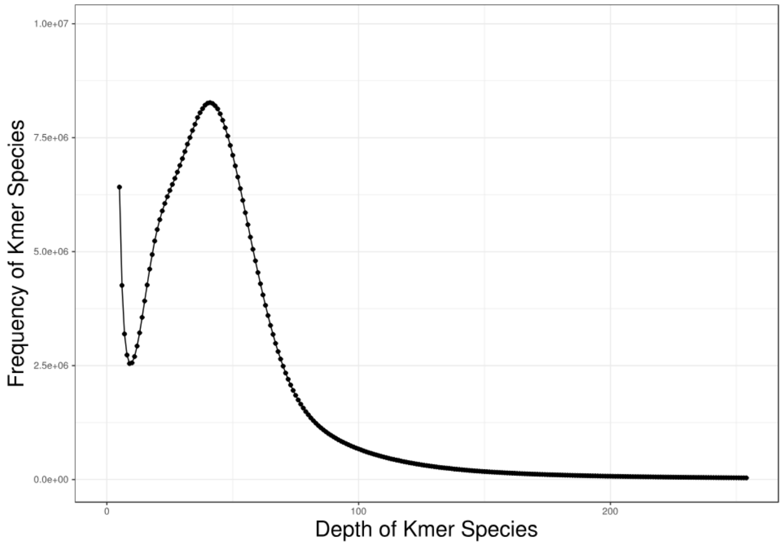

Figure 4. DNA interaction heatmap generated in HiC analysis (resolution: 500 Kb)

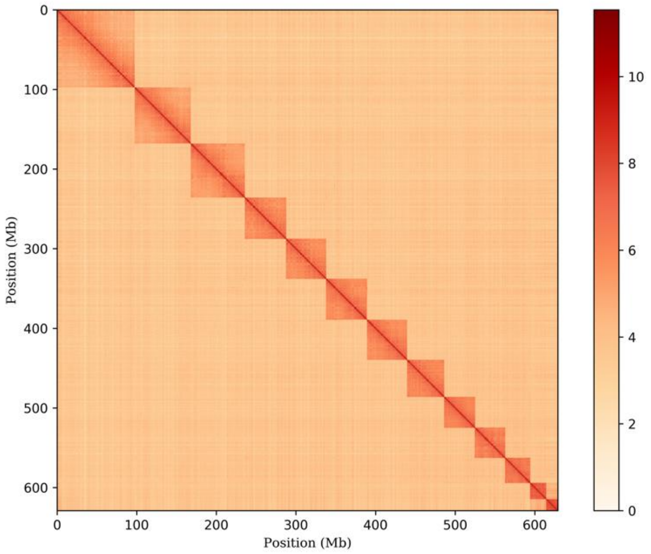

Figure 5: Genome assembly comparison of *T. rubrofasciata* with other sequenced insect genomes (*A. mellifera*, *A. pisum*, *C. lectularius*, *C. quinquefasciatus*, *D. melanogaster*, *G. buenoi*, *G. palpalis*, *H. halys*, *H. melpomene*, *H. vitripennis*, *O. fasciatus*, *R. prolixus*). The x- and y-axis represent the contig and scaffold N50s, respectively. The genomes both contig and scaffold N50s less than 2M are highlighted in black.

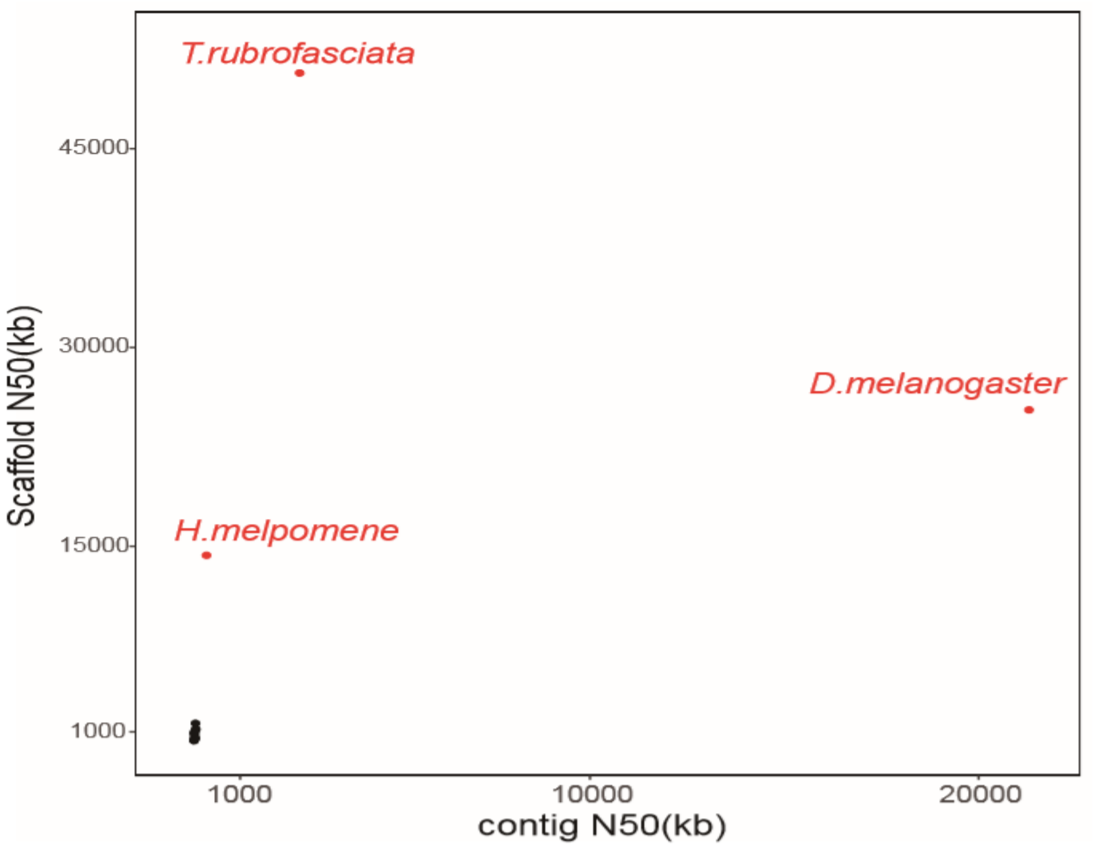

Figure 6: Length distribution comparison on total gene, CDS, exon, and intron of annotated gene models of *T. rubrofasciata* with other closely related insect species. Length distribution of total gene (A), CDS (B), exon (C), and intron (D) were compared to those of *R. prolixus*，*H. halys*，*O. fasciatus*，*C. lectularius* and *D. melanogaster*.

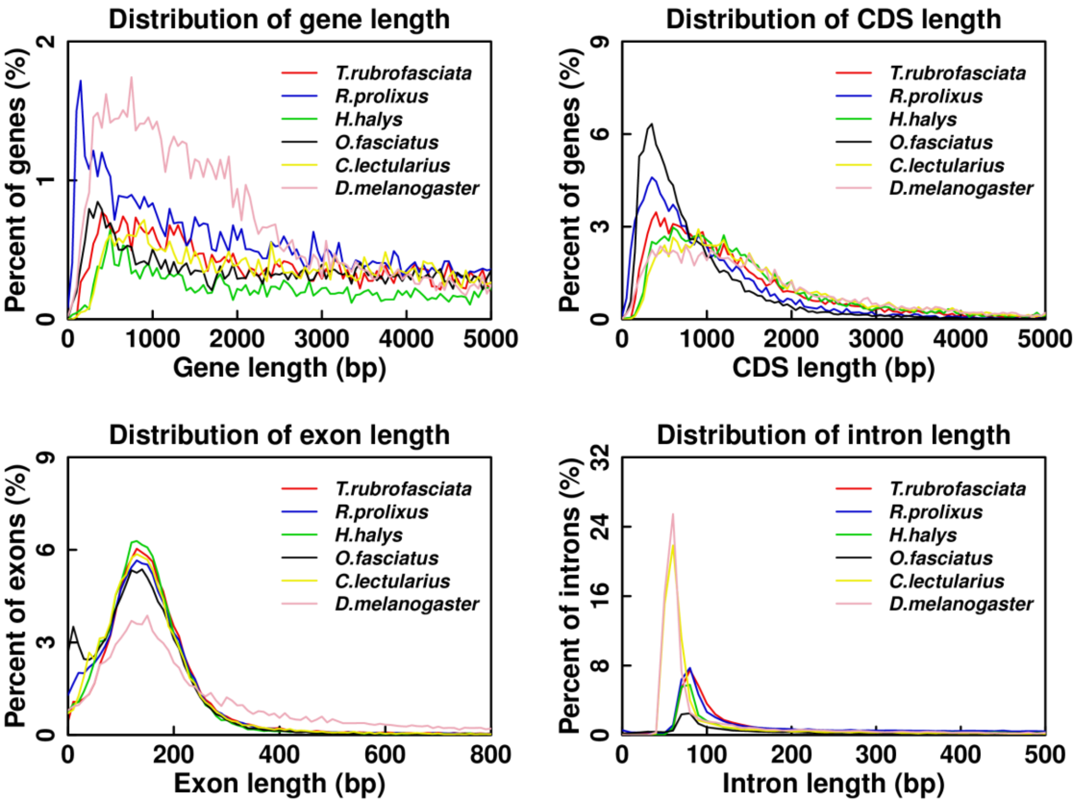

Figure 7: Phylogenetic analysis of *T. rubrofasciata* with other insect species. The estimated species divergence time (million years ago) and the 95% confidential intervals are labeled at each branch site. The divergence used for time recalibration is illuminated as red dots in the tree.

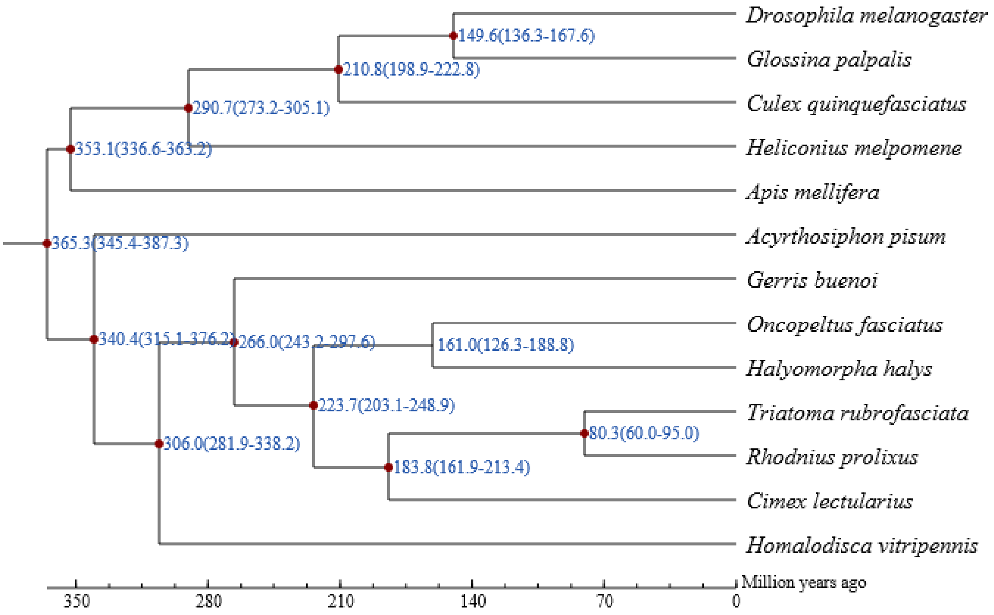

Click here to access/download
Supplementary Material
N50.xls

Personal Cover with point-to-point response to reviewers' comments
Click here to access/download;Personal Cover;point to point responses.doc

中 国 疾 病
预防控制中心
寄生虫病预防控制所
National Institute of Parasitic Disease, Chinese Center For Disease Control and Prevention
207 Ruijin Er Rd., Shanghai
Shanghai 200025, P.R. China
Website: www.ipd.org.cn

Point-to-point responses to Editors of GigaScience

Dear Editors:

Thank you very much for sending our manuscript out for review, and we would like to take advantage of this opportunity to thank the reviewers for their constructive comments.

We have read all comments by the reviewers, and have made corrections to address the issues raised by the reviewers. Most of the suggestions had either been accepted or amended accordingly in this new version. We have also prepared point-to-point responses. Our responses to each comment were written as follows, following each comment in BLUE.

We hope that our revised manuscript has effectively addressed all comments the reviewers have raised. We appreciated if you find the revised version can be published in GigaScience.

Sincerely yours,

Qin Liu (First Author)
Xiao-Nong Zhou (Corresponding author)

207 Ruijin Er Rd., Shanghai
Shanghai 200025, P.R. China
Website: www.ipd.org.cn

Replies to comments:

1. Further clarifications on how you obtained the final assembly results are required, as well as the addition of a "genome polishing" sections to further clarify the Hi-C assembly, genome evaluation with BUSCO and the curation procedure used.

Re: We have revised the sections including “Genome assembly using PacBio long reads”, “In situ Hi-C library construction and chromosome assembly using Hi-C data”, and “Genome quality evaluation”.

2. Please ensure that all raw data is accessible - Reviewer #2 was unable to access the raw data for validation.

Re: All the raw data have been released at NCBI with the following website address:https://trace.ncbi.nlm.nih.gov/Traces/study/?acc=PRJNA516044&go=go

Reviewer reports:

Reviewer #1:

The authors present a very high quality assembly of the genome for Triatoma rubrofasciata that was generated using Illumina X Ten, PacBio and Hi-C libraries. The final genome size reported was 680 Mbp with scaffold and contig N50 values that is superior to most other insect genomes reported to date. The completeness of the genome was estimated at 98% with very few duplicated regions. The genome consists of 12,695 protein coding genes of which 12,304 could be annotated. The gene models compare very well with other insect genomes with regard to gene, CDS, exon and intron length distribution. The data provided with the manuscript adequately cover all features of the study and is accessible in various databases. Some issues follow below.

1. Page 1, line 57: … BUSCO genes …

Re: Thank you. We have corrected this data in Page 1 Line 29 and Page 2 Line 1.

2. Page 3, lines 57-60: A plot of the read length distribution/ratio would be useful beyond reporting mean length.

Re: Thanks. We have added a new figure (Figure 2) to illustrate read length distribution.

3. Page 3, line 60: … mean length of reads was 8.43 Kb.

Re: Thanks. We have revised the sentence to "The mean length of these subreads was 8.43 Kb" in Page 4 Line 1.

4. Page 4, line 6: Is reference 8 the appropriate reference for the HTQC package, i.e. Yang et al., 2013?

Re: We thank the reviewer for noting this. We have corrected the reference in the manuscript.

5. Page 4, line 19-22: Independent estimation of the genome size using Kmer analysis is a nice confirmation that the PacBio assembly is correct. Can the Kmer graph be included?

Re: Thanks. We have added a new figure (Figure 3) to show Kmer analysis results.

6. Page 5, line 13: The references for LACHESIS [17] seem to be inappropriate. Reference Burton et al. 2013?

Re: We thank the reviewer for noting this. We have corrected the reference in the manuscript.

207 Ruijin Er Rd., Shanghai
Shanghai 200025, P.R. China
Website: www.ipd.org.cn

7. Page 5, line 35: It is not clear from Figure 3 how many insect genomes are compared since the majority is clustered too close to distinguish between them. Can the genomes be listed in the Figure legend?

Re: Thanks. We have added the names of the insect species in the legend of Figure 5. We have also added an additional attachment in the manuscript named N50.

中 国 疾 病
预防控制中心
寄生虫病预防控制所
National Institute of Parasitic Disease, Chinese Center For Disease Control and Prevention
207 Ruijin Er Rd., Shanghai
Shanghai 200025, P.R. China
Website: www.ipd.org.cn

name	scaffold N50(bp)	contig N50(bp)	address
A.mellifera	1208949	49814	ftp://ftp.hgsc.bcm.edu/Amellifera/Genes/Amel_4.5_OGSv3.2/
A.pisum	518546	28192	http://bipaa.genouest.org/data/public/a_pisum/
C.lectularius	1637644	42565	https://www.ncbi.nlm.nih.gov/assembly/GCF_000648675.2
C.quinquefasciatus	486756	28546	ftp://ftp.ncbi.nlm.nih.gov/genomes/all/GCF/000/209/185/GCF_000209185.1_CulPip1.0
D.melanogaster	25286936	21485538	https://www.ncbi.nlm.nih.gov/assembly/GCF_000001215.4
G.buenoi	344118	3812	ftp://ftp.hgsc.bcm.edu/I5K-pilot/Water_strider/maker_annotation/version_0.5.3/
G.palpalis	575037	21728	https://www.vectorbase.org/download/
H.halys	802423	17705	https://www.ncbi.nlm.nih.gov/assembly/GCF_000696795.2
H.melpomene	14308859	324837	http://download.lepbase.org/v4/
H.vitripennis	914009	4858	ftp://ftp.hgsc.bcm.edu/I5K-pilot/Glassy-winged_sharpshooter/maker_annotation/version_0.5.3/
O.fasciatus	339960	4047	https://usdasearch.usda.gov/search?utf8=%E2%9C%93&affiliate=usda&query=Oncopeltus+fasciatus&commit=Search
R.prolixus	1088772	34095	https://www.vectorbase.org/taxonomy/rhodnius-prolixus

207 Ruijin Er Rd., Shanghai
Shanghai 200025, P.R. China
Website: www.ipd.org.cn

8. Page 5, line 41: Not sure why sentence refers to difficulty of mollusk genome assembly? It seems as if parts of the manuscript were copied from another manuscript without updating key words or references?

Re: We thank the reviewer for spotting this. It was the unpublished work we have done in mollusk genome. We have rewritten this paragraph.

9. Page 7, lines 1-8: The phylogenetic analysis needs more information. How was the nodes calibrated for the molecular clock analysis? Include specific fossil data used for calibration. It should be noted that the date estimates for the divergence of Rhodnius/Triatoma (51-96 MYA) is much older than other molecular clock estimates (Hwang and Weirauch, 2012). In fact the upper estimate is almost as old the estimate for the higher Reduviidae as a group. The authors should at least address these differences.

Re: Thanks. We have revised the text by adding more details regarding the analysis. We added these text in Page 7 Lines 6-10: "We first obtained divergent times for all pair using the phylogenetic tree using r8s (Sanderson et al., 2003), which were used as input, together with pair-wise fossil calibration time from TimeTree (Kumer et al., 2017), to estimate species divergence time for all pairs of species in the phylogenetic tree using MCMCtree program (from PAML) (Yang et al., 1997)."

Reviewer #2: The authors sequenced a female blood-sucking insect Triatoma rubrofasciata, which is a pathogen vector of Chagas disease.

207 Ruijin Er Rd., Shanghai
Shanghai 200025, P.R. China
Website: www.ipd.org.cn

With PacBio sequencing, they reconstructed an assembly covering 99% of the 667 Mb genome, and used Hi-C analysis to reconstruct 13 haploid full-length chromosomes with a contig N50 near 3 Mb and a scaffold N50 over 50 Mb. The authors claimed a base-accuracy of 99.99%. More than 12k protein coding genes has been annotated with 97% BUSCO score that suggests a high genome completeness.

Re: Thank you very much.

The methods employed and the description in the study are mostly appropriate and standard. The integration of long-read PacBio sequencing with Hi-C analysis for chromosome reconstruction has become one of the standard pipeline for de novo genome assembly nowadays. The choice of a diploid female individual is suitable for a species without prior quality reference. Key global statistics numbers, including total length, max length and N50 of contigs and scaffolds listed in Table 2 are validated. However, there are some obvious confusion in obtaining the final assembly results. The scaffold N50 is mentioned in text several times as 51.38 Mb, while it is 50,700,875 bp in Table 2, as well as checked with the data uploaded. Similarly, contig N50 is 2.96 Mb in text, and 2,722,109 in Table 2 and data. It is unclear how the assemblies resolve from Falcon-assembly with 2,115 contigs and Hi-C assembly with 626 contigs, into the final assembly with 1,303 scaffolds. The authors should add a section of "genome polishing" between Hi-C assembly and genome evaluation with BUSCO to describe the reconciliation process, or at least mention of a curation procedure. For BUSCO genome evaluation, the authors should also specify which reference gene set was used）.

207 Ruijin Er Rd., Shanghai
Shanghai 200025, P.R. China
Website: www.ipd.org.cn

Re: Thank you. We have revised the manuscript to clarify these numbers, and have added necessary references.

1. Key global statistics numbers, including total length, max length and N50 of contigs and scaffolds listed in Table 2.

Re： Thanks. We have checked and corrected the numbers in Table 2.

2. It is unclear how the assemblies resolve from Falcon-assembly with 2,115 contigs and Hi-C assembly with 626 contigs, into the final assembly with 1,303 scaffolds

Re：Thanks. We have checked the data and rewritten those in Page 5, Lines 4-15. The assembled genome consisted of 1,030 scaffolds, which included 13 chromosomes and 1,290 unanchored scaffolds.

3. For BUSCO genome evaluation, the authors should also specify which reference gene set was used.

Re： We have added the reference gene set information in the manuscript.

4. The authors should add a section of "genome polishing" between Hi-C assembly and genome evaluation with BUSCO to describe the reconciliation process, or at least mention of a curation procedure.

Re： Thanks. We have added description about polishing in the “Genome assembly using PacBio long reads” section. We have added the reference gene set information for BUSCO analysis.

5. In addition, the unavailability of raw data and specific parameters, including the scores and thresholds for alignment and phylogenetic tree construction prevents validation. In the last section of methods on constructing the phylogenetic tree, the authors should state the source of sequences of other insects, as well as using an outgroup. Therefore, the validity of the authors' claim of species divergence time cannot be assessed.

Re: Thanks. We have submitted the data to NCBI and accession numbers are included in the revised manuscript. The source of sequences for other insects was included in a new file.
