# Supplementary material for: A chromosomal-level genome assembly for the insect vector for Chagas disease, Triatoma rubrofasciata
Source: Gigascience. 2019 Aug 19;8(8):giz089. doi: 10.1093/gigascience/giz089 (PMC6699579; doi:10.1093/gigascience/giz089)
Supplement: giz089_Response_to_Reviewer_Comments_Original_Submission [file giz089_response_to_reviewer_comments_original_submission.pdf]

Author's Response To Reviewer Comments

Close

Point-to-point responses to Editors of GigaScience

Dear Editors:

Thank you very much for sending our manuscript out for review, and we would like to take advantage of this opportunity to thank the reviewers for their constructive comments.

We have read all comments by the reviewers, and have made corrections to address the issues raised by the reviewers. Most of the suggestions had either been accepted or amended accordingly in this new version. We have also prepared point-to-point responses. Our responses to each comment were written as follows, following each comment in BLUE.

We hope that our revised manuscript has effectively addressed all comments the reviewers have raised. We appreciated if you find the revised version can be published in GigaScience.

Sincerely yours,

Qin Liu (First Author)
Xiao-Nong Zhou (Corresponding author)

Replies to comments:
1. Further clarifications on how you obtained the final assembly results are required, as well as the addition of a "genome polishing" sections to further clarify the Hi-C assembly, genome evaluation with BUSCO and the curation procedure used.
Re: We have revised the sections including "Genome assembly using PacBio long reads", "In situ Hi-C library construction and chromosome assembly using Hi-C data", and "Genome quality evaluation".

2. Please ensure that all raw data is accessible - Reviewer #2 was unable to access the raw data for validation.
Re: All the raw data have been released at NCBI with the following website address:https://trace.ncbi.nlm.nih.gov/Traces/study/?acc=PRJNA516044&go=go

Reviewer reports:
Reviewer #1:
The authors present a very high quality assembly of the genome for Triatoma rubrofasciata that was generated using Illumina X Ten, PacBio and Hi-C libraries. The final genome size reported was 680 Mbp with scaffold and contig N50 values that is superior to most other insect genomes reported to date. The completeness of the genome was estimated at 98% with very few duplicated regions. The genome consists of 12,695 protein coding genes of which 12,304 could be annotated. The gene models compare very well with other insect genomes with regard to gene, CDS, exon and intron length distribution. The data provided with the manuscript adequately cover all features of the study and is accessible in various databases. Some issues follow below.
1. Page 1, line 57: … BUSCO genes …
Re: Thank you. We have corrected this data in Page 1 Line 29 and Page 2 Line 1.
2. Page 3, lines 57-60: A plot of the read length distribution/ratio would be useful beyond reporting mean length.
Re: Thanks. We have added a new figure (Figure 2) to illustrate read length distribution.

3. Page 3, line 60: … mean length of reads was 8.43 Kb.
Re: Thanks. We have revised the sentence to "The mean length of these subreads was 8.43 Kb" in Page 4 Line 1.

4. Page 4, line 6: Is reference 8 the appropriate reference for the HTQC package, i.e. Yang et al., 2013?
Re: We thank the reviewer for noting this. We have corrected the reference in the manuscript.

5. Page 4, line 19-22: Independent estimation of the genome size using Kmer analysis is a nice confirmation that the PacBio assembly is correct. Can the Kmer graph be included?
Re: Thanks. We have added a new figure (Figure 3) to show Kmer analysis results.

6. Page 5, line 13: The references for LACHESIS [17] seem to be inappropriate. Reference Burton et al. 2013?
Re: We thank the reviewer for noting this. We have corrected the reference in the manuscript.
7. Page 5, line 35: It is not clear from Figure 3 how many insect genomes are compared since the majority is clustered too close to distinguish between them. Can the genomes be listed in the Figure legend?
Re: Thanks. We have added the names of the insect species in the legend of Figure 5. We have also added an additional attachment in the manuscript named N50.

name scaffold N50(bp) contig N50(bp) address
A.mellifera 1208949 49814 ftp://ftp.hgsc.bcm.edu/Amellifera/Genes/Amel_4.5_OGSv3.2/
A.pisum 518546 28192 http://bipaa.genouest.org/data/public/a_pisum/
C.lectularius 1637644 42565 https://www.ncbi.nlm.nih.gov/assembly/GCF_000648675.2
C.quinquefasciatus 486756 28546
ftp://ftp.ncbi.nlm.nih.gov/genomes/all/GCF/000/209/185/GCF_000209185.1_CulPip1.0
D.melanogaster 25286936 21485538 https://www.ncbi.nlm.nih.gov/assembly/GCF_000001215.4
G.buenoi 344118 3812 ftp://ftp.hgsc.bcm.edu/I5K-pilot/Water_strider/maker_annotation/version_0.5.3/
G.palpalis 575037 21728 https://www.vectorbase.org/download/
H.halys 802423 17705 https://www.ncbi.nlm.nih.gov/assembly/GCF_000696795.2
H.melpomene 14308859 324837 http://download.lepbase.org/v4/
H.vitripennis 914009 4858 ftp://ftp.hgsc.bcm.edu/I5K-pilot/Glassy-winged_sharpshooter/maker_annotation/version_0.5.3/
O.fasciatus 339960 4047
https://usdasearch.usda.gov/search?utf8=%E2%9C%93&affiliate=usda&query=Oncopeltus+fasciatus&commit=Search
R.prolixus 1088772 34095 https://www.vectorbase.org/taxonomy/rhodnius-prolixus

8. Page 5, line 41: Not sure why sentence refers to difficulty of mollusk genome assembly? It seems as if parts of the manuscript were copied from another manuscript without updating key words or references?
Re: We thank the reviewer for spotting this. It was the unpublished work we have done in mollusk genome. We have rewritten this paragraph.

9. Page 7, lines 1-8: The phylogenetic analysis needs more information. How was the nodes calibrated for the molecular clock analysis? Include specific fossil data used for calibration. It should be noted that the date estimates for the divergence of Rhodnius/Triatoma (51-96 MYA) is much older than other molecular clock estimates (Hwang and Weirauch, 2012). In fact the upper estimate is almost as old the estimate for the higher Reduviidae as a group. The authors should at least address these differences.
Re: Thanks. We have revised the text by adding more details regarding the analysis. We added these text in Page 7 Lines 6-10: "We first obtained divergent times for all pair using the phylogenetic tree using r8s (Sanderson et al., 2003), which were used as input, together with pair-wise fossil calibration time from TimeTree (Kumer et al., 2017), to estimate species divergence time for all pairs of species in the phylogenetic tree using MCMCtree program (from PAML) (Yang et al., 1997)."

Reviewer #2: The authors sequenced a female blood-sucking insect Triatoma rubrofasciata, which is a pathogen vector of Chagas disease.

With PacBio sequencing, they reconstructed an assembly covering 99% of the 667 Mb genome, and used Hi-C analysis to reconstruct 13 haploid full-length chromosomes with a contig N50 near 3 Mb and a scaffold N50 over 50 Mb. The authors claimed a base-accuracy of 99.99%. More than 12k protein coding genes has been annotated with 97% BUSCO score that suggests a high genome completeness.

Re: Thank you very much.

The methods employed and the description in the study are mostly appropriate and standard. The integration of long-read PacBio sequencing with Hi-C analysis for chromosome reconstruction has become one of the standard pipeline for de novo genome assembly nowadays. The choice of a diploid female individual is suitable for a species without prior quality reference. Key global statistics numbers, including total length, max length and N50 of contigs and scaffolds listed in Table 2 are validated. However, there are some obvious confusion in obtaining the final assembly results. The scaffold N50 is mentioned in text several times as 51.38 Mb, while it is 50,700,875 bp in Table 2, as well as checked with the data uploaded. Similarly, contig N50 is 2.96 Mb in text, and 2,722,109 in Table 2 and data. It is unclear how the assemblies resolve from Falcon-assembly with 2,115 contigs and Hi-C assembly with 626 contigs, into the final assembly with 1,303 scaffolds. The authors should add
a section of "genome polishing" between Hi-C assembly and genome evaluation with BUSCO to describe the reconciliation process, or at least mention of a curation procedure. For BUSCO genome evaluation, the authors should also specify which reference gene set was used）.
Re: Thank you. We have revised the manuscript to clarify these numbers, and have added necessary references.

1. Key global statistics numbers, including total length, max length and N50 of contigs and scaffolds listed in Table 2.
Re：Thanks. We have checked and corrected the numbers in Table 2.

2. It is unclear how the assemblies resolve from Falcon-assembly with 2,115 contigs and Hi-C assembly with 626 contigs, into the final assembly with 1,303 scaffolds
Re：Thanks. We have checked the data and rewritten those in Page 5, Lines 4-15. The assembled genome consisted of 1,030 scaffolds, which included 13 chromosomes and 1,290 unanchored scaffolds.

3. For BUSCO genome evaluation, the authors should also specify which reference gene set was used.
Re：We have added the reference gene set information in the manuscript.

4. The authors should add a section of "genome polishing" between Hi-C assembly and genome evaluation with BUSCO to describe the reconciliation process, or at least mention of a curation procedure.
Re：Thanks. We have added description about polishing in the “Genome assembly using PacBio long reads” section. We have added the reference gene set information for BUSCO analysis.

5. In addition, the unavailability of raw data and specific parameters, including the scores and thresholds for alignment and phylogenetic tree construction prevents validation. In the last section of methods on constructing the phylogenetic tree, the authors should state the source of sequences of other insects, as well as using an outgroup. Therefore, the validity of the authors' claim of species divergence time cannot be assessed.
Re: Thanks. We have submitted the data to NCBI and accession numbers are included in the revised manuscript. The source of sequences for other insects was included in a new file.

Close
