# Supplementary material for: A chromosomal-level genome assembly for the insect vector for Chagas disease, Triatoma rubrofasciata
Source: Gigascience. 2019 Aug 19;8(8):giz089. doi: 10.1093/gigascience/giz089 (PMC6699579; doi:10.1093/gigascience/giz089)
Supplement: giz089_Response_to_Reviewer_Comments_Revision_1 [file giz089_response_to_reviewer_comments_revision_1.pdf]

Author's Response To Reviewer Comments

Close

Point-to-point responses to Editors of GigaScience

Dear Editors:

Thank you very much.

We have read all comments word by word, along with those corrections in the edited manuscript. The suggestions had been accepted and amended carefully in this new version. All questions had been answered in this point-to-point response, and our response to each comment wass written as follows, following each comment in BLUE.

We should be appreciated if you could take the revised version consideration to be published in GigaScience.

Sincerely yours,

Qin Liu (First Author)
Xiao-Nong Zhou (Corresponding author)

Replies to comments:
In addition, please register any new software application in the SciCrunch.org database to receive a RRID (Research Resource Identification Initiative ID) number, and include this in your manuscript. This will facilitate tracking, reproducibility and re-use of your tool.
Re：Thank you. No new software application was needed to register in this manuscript. The reference of the GCE software was inserted in Page 4 line 13.

Reviewer reports:
Reviewer #1: Most reviewer issues were addressed satisfactorily. Some minor issues is suggested below.

Page 4, line 2: ... distribution/ratio is showed in Figure 2.
Re：Thank you. “showing” was changed to “showed” in Page4, line2.

Figure 3: Unit of X-axes? How does this relate to estimated genome size?
Re：Thank you. The name and unit of X-axes in Figure 3 was changed in the new version. The genome size of T. rubrofasciata was estimated by using the Kmer-based method in GCE software (Liu B , Shi Y , Yuan J , et al. Estimation of genomic characteristics by analyzing k-mer frequency in de novo genome projects. Quantitative Biology, 2013, 35(s 1–3):62-67). We calculated and plotted the 17-mer depth distribution in Figure 3. The X-axes was the kmer count, means the peak frequency of 17-mers. The peak frequency was estimated around 41 and the genome size of T. rubrofasciata was estimated to be 757 Mb on the basis of the formula “G = N17−mer/D17−mer”, where the N17−mer was the number of 17-mers, D17−mer denoted the peak depth of 17-mers estimated, and G represented the estimated genome size.

Page 5, line 28: Please note that the last sentence of this paragraph still refer to the difficulty of the

mollusk genome assembly. This paragraph also contained numerous small errors. Please see suggested modified paragraph below:
Re：Thank you. This paragraph was changed as the reviewer's suggestion.

Reviewer #2: The authors have sufficiently addressed all of my concerns.
Re：Thank you.

Additional:
The pictures of female T. rubrofasciata were changed in Figure 1.

Close
