# Supplementary material for: A chromosomal-level genome assembly for the insect vector for Chagas disease, Triatoma rubrofasciata
Source: Gigascience. 2019 Aug 19;8(8):giz089. doi: 10.1093/gigascience/giz089 (PMC6699579; doi:10.1093/gigascience/giz089)
Supplement: giz089_Reviewer_1_Report_Original_Submission — Ben Mans -- 2/25/2019 Reviewed [file giz089_reviewer_1_report_original_submission.pdf]

Reviewer Report

Title: A Chromosomal-Level Genome Assembly for the insect vector for Chagas disease, Triatoma rubrofasciata

Version: Original Submission **Date:** 2/25/2019

Reviewer name: Ben Mans

Reviewer Comments to Author:

The authors present a very high quality assembly of the genome for Triatoma rubrofasciata that was generated using Illumina X Ten, PacBio and Hi-C libraries. The final genome size reported was 680 Mbp with scaffold and contig N50 values that is superior to most other insect genomes reported to date. The completeness of the genome was estimated at 98% with very few duplicated regions. The genome consists of 12,695 protein coding genes of which 12,304 could be annotated. The gene models compare very well with other insect genomes with regard to gene, CDS, exon and intron length distribution. The data provided with the manuscript adequately cover all features of the study and is accessible in various databases. Some issues follow below.
Page 1, line 57: … BUSCO genes …
Page 3, lines 57-60: A plot of the read length distribution/ratio would be useful beyond reporting mean length.
Page 3, line 60: … mean length of reads was 8.43 Kb.
Page 4, line 6: Is reference 8 the appropriate reference for the HTQC package, i.e. Yang et al., 2013?
Page 4, line 19-22: Independent estimation of the genome size using Kmer analysis is a nice confirmation that the PacBio assembly is correct. Can the Kmer graph be included?
Page 5, line 13: The references for LACHESIS [17] seem to be inappropriate. Reference Burton et al. 2013?
Page 5, line 35: It is not clear from Figure 3 how many insect genomes are compared since the majority is clustered too close to distinguish between them. Can the genomes be listed in the Figure legend?
Page 5, line 41: Not sure why sentence refers to difficulty of mollusk genome assembly? It seems as if parts of the manuscript were copied from another manuscript without updating key words or references?
Page 7, lines 1-8: The phylogenetic analysis needs more information. How was the nodes calibrated for the molecular clock analysis? Include specific fossil data used for calibration. It should be noted that the date estimates for the divergence of Rhodnius/Triatoma (51-96 MYA) is much older than other molecular clock estimates (Hwang and Weirauch, 2012). In fact the upper estimate is almost as old the estimate for the higher Reduviidae as a group. The authors should at least address these differences.

Level of Interest

Please indicate how interesting you found the manuscript: Choose an item.

Quality of Written English

Please indicate the quality of language in the manuscript: Choose an item.

Declaration of Competing Interests

Please complete a declaration of competing interests, considering the following questions:

- Have you in the past five years received reimbursements, fees, funding, or salary from an organisation that may in any way gain or lose financially from the publication of this manuscript, either now or in the future?
- Do you hold any stocks or shares in an organisation that may in any way gain or lose financially from the publication of this manuscript, either now or in the future?
- Do you hold or are you currently applying for any patents relating to the content of the manuscript?
- Have you received reimbursements, fees, funding, or salary from an organization that holds or has applied for patents relating to the content of the manuscript?
- Do you have any other financial competing interests?
- Do you have any non-financial competing interests in relation to this paper?

If you can answer no to all of the above, write 'I declare that I have no competing interests' below. If your reply is yes to any, please give details below.

I declare that I have no competing interests

I agree to the open peer review policy of the journal. I understand that my name will be included on my report to the authors and, if the manuscript is accepted for publication, my named report including any attachments I upload will be posted on the website along with the authors' responses. I agree for my report to be made available under an Open Access Creative Commons CC-BY license (http://creativecommons.org/licenses/by/4.0/). I understand that any comments which I do not wish to be included in my named report can be included as confidential comments to the editors, which will not be published.

I agree to the open peer review policy of the journal

To further support our reviewers, we have joined with Publons, where you can gain additional credit to further highlight your hard work (see: https://publons.com/journal/530/gigascience). On publication of this paper, your review will be automatically added to Publons, you can then choose whether or not to claim your Publons credit. I understand this statement.

Yes Choose an item.
