# Supplementary material for: A chromosomal-level genome assembly for the insect vector for Chagas disease, Triatoma rubrofasciata
Source: Gigascience. 2019 Aug 19;8(8):giz089. doi: 10.1093/gigascience/giz089 (PMC6699579; doi:10.1093/gigascience/giz089)
Supplement: giz089_Reviewer_2_Report_Original_Submission — Ting-Fung Chan, PhD -- 3/9/2019 Reviewed [file giz089_reviewer_2_report_original_submission.pdf]

Reviewer Report

Title: A Chromosomal-Level Genome Assembly for the insect vector for Chagas disease, Triatoma rubrofasciata

Version: Original Submission **Date:** 3/9/2019

Reviewer name: Ting-Fung Chan, PhD

Reviewer Comments to Author:

The authors sequenced a female blood-sucking insect Triatoma rubrofasciata, which is a pathogen vector of Chagas disease.
With PacBio sequencing, they reconstructed an assembly covering 99% of the 667 Mb genome, and used Hi-C analysis to reconstruct 13 haploid full-length chromosomes with a contig N50 near 3 Mb and a scaffold N50 over 50 Mb. The authors claimed a base-accuracy of 99.99%. More than 12k protein coding genes has been annotated with 97% BUSCO score that suggests a high genome completeness.
The methods employed and the description in the study are mostly appropriate and standard. The integration of long-read PacBio sequencing with Hi-C analysis for chromosome reconstruction has become one of the standard pipeline for de novo genome assembly nowadays. The choice of a diploid female individual is suitable for a species without prior quality reference. Key global statistics numbers, including total length, max length and N50 of contigs and scaffolds listed in Table 2 are validated.
However, there are some obvious confusion in obtaining the final assembly results. The scaffold N50 is mentioned in text several times as 51.38 Mb, while it is 50,700,875 bp in Table 2, as well as checked with the data uploaded. Similarly, contig N50 is 2.96 Mb in text, and 2,722,109 in Table 2 and data. It is unclear how the assemblies resolve from Falcon-assembly with 2,115 contigs and Hi-C assembly with 626 contigs, into the final assembly with 1,303 scaffolds. The authors should add a section of "genome polishing" between Hi-C assembly and genome evaluation with BUSCO to describe the reconciliation process, or at least mention of a curation procedure. For BUSCO genome evaluation, the authors should also specify which reference gene set was used.
In addition, the unavailability of raw data and specific parameters, including the scores and thresholds for alignment and phylogenetic tree construction prevents validation. In the last section of methods on constructing the phylogenetic tree, the authors should state the source of sequences of other insects, as well as using an outgroup. Therefore, the validity of the authors' claim of species divergence time cannot be assessed.

Level of Interest

Please indicate how interesting you found the manuscript: Choose an item.

Quality of Written English

Please indicate the quality of language in the manuscript: Choose an item.

Declaration of Competing Interests

Please complete a declaration of competing interests, considering the following questions:

- Have you in the past five years received reimbursements, fees, funding, or salary from an organisation that may in any way gain or lose financially from the publication of this manuscript, either now or in the future?
- Do you hold any stocks or shares in an organisation that may in any way gain or lose financially from the publication of this manuscript, either now or in the future?
- Do you hold or are you currently applying for any patents relating to the content of the manuscript?
- Have you received reimbursements, fees, funding, or salary from an organization that holds or has applied for patents relating to the content of the manuscript?
- Do you have any other financial competing interests?
- Do you have any non-financial competing interests in relation to this paper?

If you can answer no to all of the above, write 'I declare that I have no competing interests' below. If your reply is yes to any, please give details below.

I declare that I have no competing interests

I agree to the open peer review policy of the journal. I understand that my name will be included on my report to the authors and, if the manuscript is accepted for publication, my named report including any attachments I upload will be posted on the website along with the authors' responses. I agree for my report to be made available under an Open Access Creative Commons CC-BY license (http://creativecommons.org/licenses/by/4.0/). I understand that any comments which I do not wish to be included in my named report can be included as confidential comments to the editors, which will not be published.

I agree to the open peer review policy of the journal

To further support our reviewers, we have joined with Publons, where you can gain additional credit to further highlight your hard work (see: https://publons.com/journal/530/gigascience). On publication of this paper, your review will be automatically added to Publons, you can then choose whether or not to claim your Publons credit. I understand this statement.

Yes Choose an item.
